# Concise Overview of Glypromate Neuropeptide Research: From Chemistry to Pharmacological Applications in Neurosciences

**DOI:** 10.1021/acschemneuro.2c00675

**Published:** 2023-02-03

**Authors:** Sara C. Silva-Reis, Ivo E. Sampaio-Dias, Vera M. Costa, Xavier Cruz Correia, Hugo F. Costa-Almeida, Xerardo García-Mera, José E. Rodríguez-Borges

**Affiliations:** † LAQV/REQUIMTE, Department of Chemistry and Biochemistry, Faculty of Sciences, University of Porto, 4169-007 Porto, Portugal; ‡ UCIBIO/REQUIMTE, Laboratory of Toxicology, Faculty of Pharmacy, University of Porto, 4050-313 Porto, Portugal; § Associate Laboratory i4HB, Institute for Health and Bioeconomy, Faculty of Pharmacy, University of Porto, 4050-313 Porto, Portugal; ∥ Department of Organic Chemistry, Faculty of Pharmacy, University of Santiago de Compostela, E-15782 Santiago de Compostela, Spain

**Keywords:** Glypromate, Gly-Pro-Glu, GPE, neurodegenerative diseases, peptidomimetics, Trofinetide

## Abstract

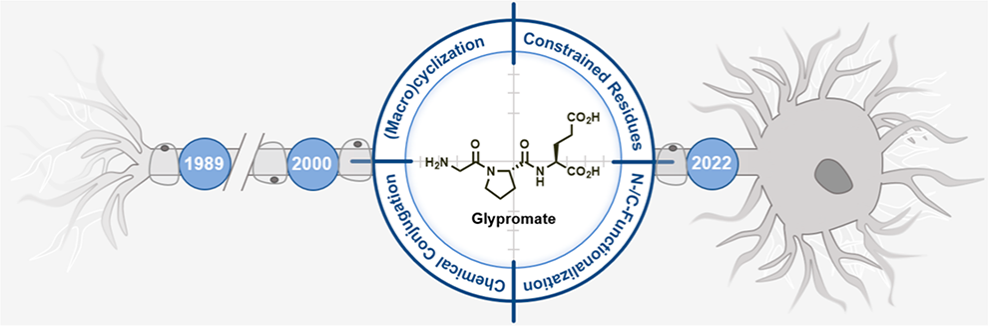

Neurodegenerative diseases of the central nervous system (CNS) pose a serious health concern worldwide, with a particular incidence in developed countries as a result of life expectancy increase and the absence of restorative treatments. Presently, treatments for these neurological conditions are focused on managing the symptoms and/or slowing down their progression. As so, the research on novel neuroprotective drugs is of high interest. Glypromate (glycyl-l-prolyl-l-glutamic acid, also known as GPE), an endogenous small peptide widespread in the brain, holds great promise to tackle neurodegenerative diseases such as Parkinson’s, Alzheimer’s, and Huntington’s, s well as other CNS-related disorders like Rett and Down’s syndromes. However, the limited pharmacokinetic properties of Glypromate hinder its clinical application. As such, intense research has been devoted to leveraging the pharmacokinetic profile of this neuropeptide. This review aims to offer an updated perspective on Glypromate research by exploring the vast array of chemical derivatizations of more than 100 analogs described in the literature over the past two decades. The collection and discussion of the most relevant structure–activity relationships will hopefully guide the discovery of new Glypromate-based neuroprotective drugs.

## 1 Introduction

**Figure 1:**
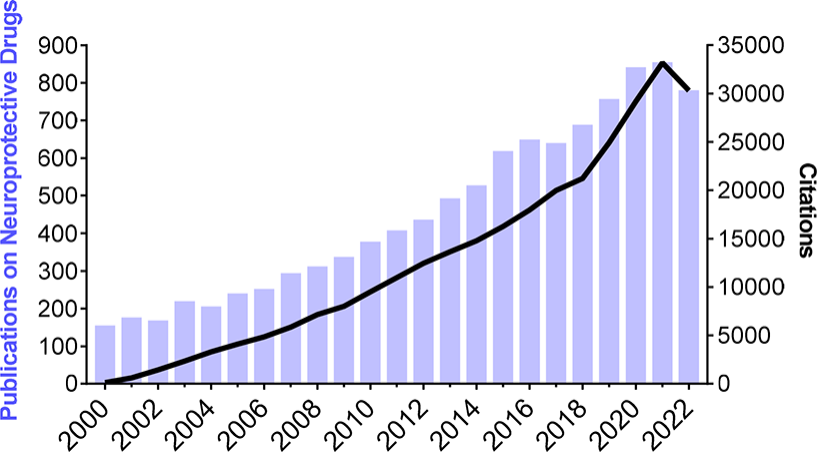
Publications and citations on the topic of neuroprotective drugs research for the 2000 to 2022 time period. Data were obtained from the Web of Science for the query “neuroprotective” (search: article titles). Both publication and citation numbers trend upward indicating an increase in interest in this field overtime.

The topic of central nervous system (CNS) disorders, in which neurodegenerative diseases, chronic neurodegeneration, and traumatic brain injury are included, garners a fair amount of consideration from the scientific community. At present, the lack of effective pharmaceuticals to treat these neurological conditions is a great unmet medical need. The research on neuroprotective drugs has steadily increased over the past decades as corroborated by the rising number of primary and secondary publications on this topic (Figure 1), with pharmaceutical companies focusing their efforts on the development of small bioactive molecules, such as the Glypromate neuropeptide.

Peptide-based neurotherapeutics have emerged as a promising strategy to tackle CNS-related disorders due to their high activity, specificity, and affinity for biological targets. However, due to their intrinsic peptide nature, this class of compounds exhibits pharmacokinetic drawbacks (e.g., high lability toward proteases), hindering their clinical application. Peptide analogs targeting the CNS producing a similar or improved effect while circumventing the drawbacks associated with the parent neuropeptides are therefore of high interest in neurosciences. This approach has led to the *de novo* discovery of peptide derivatives such as Trofinetide. Despite the undeniable therapeutical potential and relevance of Glypromate neuropeptide in this regard, to the best of our knowledge, there are three reviews in the literature on the insulin-like growth factor-1 (IGF-1) and its metabolites,^1−3^ and only one brief minireview exclusively devoted to research on Glypromate neuropeptide, covering structural modifications and biological activities of its analogs (as of 2012).^4^ Therefore, this review aims to provide the big picture on Glypromate research delivering a comprehensive, critical, and integrative overview ranging from organic synthesis (covering the most representative structural modifications and chemical strategies) to the pharmacological and biological applications of this neuropeptide and structure-related compounds. Herein, English-written peer-reviewed articles and patents dated from January 2000 to December 2022 were considered and revised after a survey at the Scifinder software using the following keywords: “Glypromate” and “glycyl-l-prolyl-l-glutamic acid”. Nonetheless, seminal studies and other relevant documents on Glypromate research prior to 2000 were also included for integrative and comprehensive purposes. This review is organized in logical sections such as biosynthesis and metabolism of Glypromate, recent developments and advances in chemical methodologies for the assembly of Glypromate and structure-related analogs, detailed analysis of the main chemical modifications performed at each amino acid residue, cyclization and macrocyclization strategies, as well as chemical conjugation with biomolecules and other pertinent chemical derivatizations, highlighting the main structure–activity relationships reported in the literature. The interplay between these approaches and the challenges associated with Glypromate research are analyzed to contribute to further progress in the development of new Glypromate-based neuroprotective drugs.

## 2 Glypromate: Chemical and Biochemical Properties

### 2.1 Biosynthesis and Pharmacokinetics

**Figure 2:**
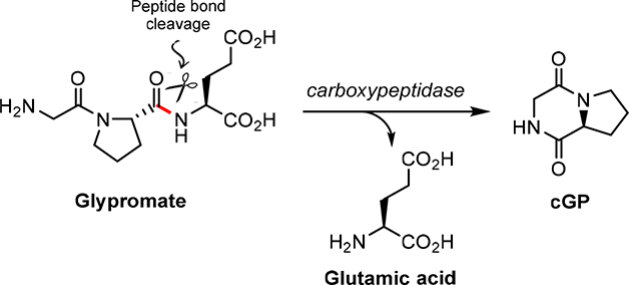
Metabolization of Glypromate by carboxypeptidases affording cGP, a neuroprotective metabolite, and glutamic acid.

Glypromate, formally known as glycyl-l-propyl-l-glutamic acid (GPE, according to the one-letter system of peptides, Figure 2) is an endogenous neuropeptide derived from the IGF-1 (a potent neurotrophic and antiapoptotic factor produced in the brain that plays an important role in its normal development, metabolism, and recovery from injury).^5^ Glypromate corresponds to the N-terminal sequence of IGF-1, being produced after IGF-1 metabolism by an acid protease^6^ along with des-*N*(1-3)-IGF-1, the truncated form of IGF-1.^7^ This neuropeptide is metabolized by carboxypeptidases affording glutamic acid along with cyclo-glycyl-l-proline (cGP, Figure 2), a nootropic compound with neuroprotective activities within the CNS.^1,8^ Despite being an interesting bioactive compound, cGP will not be covered in this review due to the limited biological studies reported in the literature.

Interestingly, while in human plasma Glypromate exhibits a half-life of over 30 min,^1^ in the plasma of adult male Wistar rats, the half-life of Glypromate (3 mg kg^–1^) was found to be extremely short [less than 2 min after a single-bolus intravenous administration and less than 4 min after intraperitoneal (ip) administration],^1^ denoting significative interspecies differences in peptidase activity. In plasma, acid peptidase inhibitors pepstatin-A and bestatin were shown to increase the half-life of Glypromate, by reducing the extension of its metabolism.^9^ In contrast, Glypromate can be detected in the cerebrospinal fluid (CSF) of these animals up to 40 min after ip administration.^9^ Despite the rapid plasma clearance of this neuropeptide, full clearance of Glypromate in the CSF requires 60 min after ip administration.^9^ Interestingly, the presence of endopeptidase inhibitors, such as 4-(2-aminoethyl)benzenesulfonyl fluoride, was able to reduce Glypromate metabolism in brain tissues, being detected after 3 h of intracerebroventricular infusion of 1 μC [^3^H]-Glypromate (adult male Wistar rats).^9^ In fact, other studies corroborate that this neuropeptide is extensively metabolized in both plasma^10^ and the brain, being a target of enzymatic degradation.^1,11^

To reduce the enzymatic proteolysis of this neuropeptide, besides the use of protease inhibitors already described, Thomas and co-workers patented the use of anti-Glypromate antibodies,^12^ as a strategy to extend the half-life of Glypromate both *in vitro* and *in vivo*.^13^ However, their pharmacological use is blunted as the passive immunization against Glypromate results in loss of neuroprotective activity.^13^ Nevertheless, anti-Glypromate antibodies may find application in biochemical and biomolecular analysis to aid in the discovery of Glypromate biological targets (e.g., competition assays) or its detection by immunofluorescence/staining.

Using adult Wistar rats, it was shown that Glypromate can reach the CNS after brain injury.^14,15^ This can be explained by the activation of gelatinase matrix metalloproteinase MMP-2 (gelatinase A) and MMP-9 (gelatinase B) in injured tissues, which digest the extracellular matrix disrupting the organization of the cells and facilitating the permeabilization of Glypromate across the blood–brain barrier (BBB).^16^ In this sense, Glypromate can find application as a neuroprotective agent in brain injuries (e.g., traumatic brain injury), as it is conditionally able to reach the affected brain regions.^14^

In fact, the small size and relative stability of both Glypromate and cGP in the CNS make them great candidates for therapeutic purposes such as neuropathological conditions.^6^ However, with the reduced half-life of Glypromate in plasma, it is essential to understand how to improve its biochemical resistance toward serum proteases for the development of stable Glypromate-based drugs.^8^ Although theoretical continuous administration of Glypromate by intravenous infusion could be used to afford sustained neuroprotective effects,^10^ less invasive alternatives are mandatory, for example, through the development of orally compatible Glypromate analogs with improved pharmacokinetic profiles.

### 2.2 Biological and Pharmacological Applications

The first report on the biological and pharmacological properties of Glypromate was published in 1989 by Sara and co-workers.^17^ In this seminal work, the authors found that synthetic Glypromate promoted the potentiation of the potassium-induced release of [^3^H]-acetylcholine from parietal cortex slices of adult rats at 10^–10^ M (*p* < 0.05) and 10^–8^ M (*p* < 0.01) and a mild increase in the release of [^3^H]-dopamine from striatum at 10^–5^ M (*p* < 0.05) and 10^–4^ M (*p* < 0.01).^17^ In the same study, Sara and co-workers also demonstrated that Glypromate can bind to *N*-methyl-d-aspartate (NMDA) receptors using rat synaptosomal membranes, in which the glutamate residue plays a crucial role, whereas the glycine residue is important for potency.^17^ However, while interaction with the NMDA receptors may justify the increase in dopamine release, a different mechanism of action is hinted for the release of acetylcholine in the cortex.^17^

Later on, Ikeda and co-workers explored the mitogenic effect of Glypromate in human retinal glial cells (Müller cells).^18^ It was found that Glypromate significantly increased the number of cells upon incubation for 2 days (10–1000 μM, *p* = 0.002).^18^ The results showed a concentration-dependent tendency, with the maximal stimulation at 500 μM, while the half-maximal effective concentration (EC_50_) was determined to be approximately 50 μM.^18^ Incubation of Müller cells with Glypromate for different time periods evidenced that exposure to Glypromate for 18 h or less did not result in significant cell growth. However, the proliferation of the cells increased significantly after a 24-h or more (*p* ≤ 0.001).^18^ In order to study the mechanism behind this mitogenic activity, Müller cells were incubated with Glypromate in the presence of d-2-amino-5-phosphonovalerate (APV, an NMDA receptors antagonist), MK-801 (a noncompetitive NMDA receptor antagonist), or CNQX (6-cyano-7-nitroquinoxaline-2,3-dione, a blocker of non-NMDA glutamate receptors). In the presence of APV (100 μM) and MK-801 (1 μM), the cell growth was significantly lower when compared to Glypromate alone (*p* < 0.001), while CNQX did not exhibit significant inhibition of cell growth.^18^ Thus, the NMDA receptors appear to be responsible for the mitogenic activity of Glypromate in Müller cells.^18^ Moreover, des-*N*(1-3)-IGF-1 was also tested and it was found to be a potent mitogen with an EC_50_ around 130 pM, being up to 5 times more potent than IGF-1.^18^ The mitogenic effects of des-*N*(1-3)-IGF-1 were found to be the result of the interaction with IGF-1 receptors.^18^ Moreover, an additive mitogenic effect was observed when both Glypromate and des-*N*(1-3)-IGF-1 were tested in combination.^18^

It was also verified that Glypromate does not modulate DNA synthesis mediated by the brain IGF-1 receptor (assessed by [^3^H]-thymidine incorporation) nor does it interact with nicotinic and muscarinic receptors (data not shown).^17^

These discoveries paved the way for subsequent studies disclosing the neuroprotective effects and promising therapeutical applications of Glypromate in different animal models of neurodegenerative diseases.^19^

Later, Alexi and co-workers performed *in vivo* studies using male Wistar rats lesioned with quinolinic acid as a model of Huntington’s disease.^20^ The lesioned animals received daily injections of Glypromate (0.3 μg μL^–1^/day) or phosphate buffered saline (PBS, pH = 7.4) for 7 days.^20^ In this study, several striatal γ-aminobutyric acid (GABA) neuronal phenotypes were analyzed for evidence of neuroprotection and identified by immunohistochemical techniques.^20^ For projection neurons, GAD_67_ and calbindin phenotypes were studied. It was observed that in animals unilaterally lesioned with quinolinic acid, the number of immunostained GAD_67_ neurons decreased to 42.3 ± 3.9% while immunostained calbindin exhibited a similar pattern.^20^ Glypromate administration significantly increased the survival of calbindin neurons from 52.8 ± 2.2% to 90.6 ± 3.8% (*p* < 0.01). However, these neurons lost their ability to express GAD_67_.^20^ For interneurons, nicotinamide-adenine dinucleotide phosphate diaphorase (NADPHd), parvalbumin, and calretinin phenotypes were investigated. In this set of analyses, Glypromate was able to significantly rescue the NADPHd levels from 40.6 ± 1.8% to 71.3 ± 5.2% (*p* < 0.01), but no effect was observed for the parvalbumin and calretinin phenotypes as the neuronal survival remained practically unchanged in both the control and Glypromate-treated animals (around 55%).^20^ Striatal cholinergic interneurons immunostained for choline acetyltransferase (ChAT) were also studied. It was observed that Glypromate treatment showed a significant rescue effect on cholinergic interneurons upon quinolinic acid injury by increasing cell viability from 58.2 ± 2.0% to 85.3 ± 6.0% (*p* < 0.01).^20^ As such, Glypromate evidenced selective neuroprotective activity for a variety of GABAergic and cholinergic striatal neurons in an animal model of Huntington’s disease.

*In vitro* studies show that Glypromate activates glutamate receptors such as NMDA receptors by interaction with the glutamate-binding site (without significant binding at the glycine site)^21^ and the α-amino-3-hydroxy-5-methyl-4-isoxazolepropionic acid (AMPA) receptors.^21^ Considering the activity of Glypromate on AMPA receptors, the co-use of Glypromate and AMPA was patented by Krissansen and co-workers in 2002 for the treatment of demyelinating diseases of the CNS.^22^

Using rat hippocampal organotypic cultures, Saura’s group showed that when Glypromate (1–100 μM) is preincubated 30 min before NMDA-induced injury (100 μM), a statistical (*p* < 0.01) prevention of neuronal death is achieved.^23^ In the same study, the mechanism of action of Glypromate was also investigated, and the data gathered suggest that this neuropeptide may interact with several components of the IGF system or several NMDA receptor subtypes since when one of these receptors is stimulated with exogenous Glypromate, the progress of neuronal death is prevented.^23^ Although Glypromate can protect hippocampal neurons from NMDA-induced neuronal toxicity,^23^ this neuroprotective activity is not related to glutamate receptor binding since some analogs that had a high affinity for the glutamate receptors did not exhibit neuroprotection in cultured hippocampal neurons.^24^

Furthermore, this neuropeptide was able to partially block somatostatin (SRIF) depletion induced by amyloid β (Aβ) in the temporal cortex.^8,25^ Some studies show that Glypromate mimics the effects of IGF-1 on the SRIF system through a mechanism other than Aβ clearance such as modulation of calcium and glycogen synthase kinase 3β (GSK-3β) signaling pathway.^8,25^ These results are relevant since the reduction of SRIF is implicated in Alzheimer’s disease (AD), as does the GSK-3β.^8,25^

In addition, Glypromate demonstrated a positive effect on the regulation of the proliferation and survival of mouse embryonic neural stem cells *in vitro* by activation of the extracellular signal-regulated kinase (ERK) and phosphoinositide 3-kinase (PI3K) pathways, via NMDA receptor activation.^26^ It was also observed that Glypromate reduces the basal activity of some common kinases involved in Down’s syndrome like ERK, p38 mitogen-activated protein kinase (p38 MAPK), and GSK-3β using HTK cells (derived from the hippocampus of trisomy 16 mouse fetus),^27^ indicating that Glypromate can provide lower signaling in these neurons.

Moreover, this neuropeptide stimulates both dopamine and acetylcholine release in rat cortical slices without interacting with IGF-1 receptors;^28^ thereby, the administration of Glypromate as a pharmacological therapy for preventing the loss of dopaminergic neurons in Parkinson’s disease (PD) was extensively explored and patented by Gluckman’s group.^29−33^ The effect of Glypromate on neurodegeneration was also demonstrated in male Wistar rats using the neurotoxin 6-hydroxydopamine (6-OHDA) as an animal model of PD. With only a single dose administered intracerebroventricularly (3 μg μL^–1^), Glypromate was able to prevent the loss of tyrosine hydroxylase (TH) immunopositive neurons in the substantia nigra,^34^ the reduction of apomorphine-induced rotations, and long-term forelimb akinesia.^35,36^

*In vivo,* Glypromate reduces hypoxic–ischemic (HI) injury in adult male Wistar rats.^37^ Some authors like Guan and co-workers suggested that this neuroprotective effect may result from the inhibition of caspase 3 and non-caspase-3 activated apoptotic pathways.^38^

This neuropeptide also exhibited selective neuroprotection among different neuronal phenotypes preventing the loss of choline acetyltransferase-positive cholinergic neurons and glutamic acid decarboxylase (GAD) and somatostatin immunopositive GABA interneurons.^37^

Glypromate was additionally tested in young adults and aged male Wistar rats after ischemic–reperfusion injury induced by cardiac arrest followed by resuscitation.^39^ Intravenous infusion of Glypromate 3 h after the injury reduced the overall damage scores, as seen by a significant reduction of the apoptotic neurons in both young and aged rats.^39^ Overall, the data suggest that the neuroprotection promoted by this neuropeptide is not age-selective.^39^

Glypromate has also been tested in methyl cytosine-phosphate group-guanine binding protein 2 mutant mice, a ubiquitous protein that regulates gene expression which is particularly abundant in brain cells. In that study, the administration of Glypromate led to an increase in the lifespan and improvement of locomotor, respiratory, and cardiac functions,^40^ being considered a promising drug for Rett syndrome, in which these factors are compromised.^40−42^

Despite the broad range of biological activities and potential therapeutical applications of Glypromate, its precise mechanisms of action remain unclear.^43^ Marinelli and co-workers proposed that this neuropeptide may be involved in the modulation of inflammation, promotion of astrocytosis, inhibition of apoptosis, and vascular remodeling.^43^

The potential of Glypromate was explored in clinical trials by Neuren Pharmaceuticals Ltd. for cognitive impairment in patients undergoing cardiac surgery with cardiopulmonary bypass.^44^ However, in December 2008, the company discontinued further development after Glypromate failed to show a meaningful neuroprotective effect in that population,^44^ with no significant difference observed during the study (12 weeks) between patients receiving a placebo from those receiving Glypromate on either change in cognitive score or activities of daily living.^44^

As so, the research on Glypromate-based peptidomimetics is of utmost importance to enlighten the mechanism of action of this neuropeptide and for the development of neurotherapeutics with improved neuroprotective activity and pharmacokinetic profiles.

### 2.3 Synthesis of Glypromate and Structure-Related Analogs

Glypromate and its analogs are generally synthesized by common strategies in peptide chemistry, such as solid-phase peptide synthesis (SPPS) and classical liquid-phase peptide synthesis (LPPS). In this review, two interesting advances in SPPS and LPPS methodologies designed specifically to boost the research on Glypromate and structure-related peptidomimetics are highlighted.

#### 2.3.1 SPPS-Based Approach

**Scheme 1:**
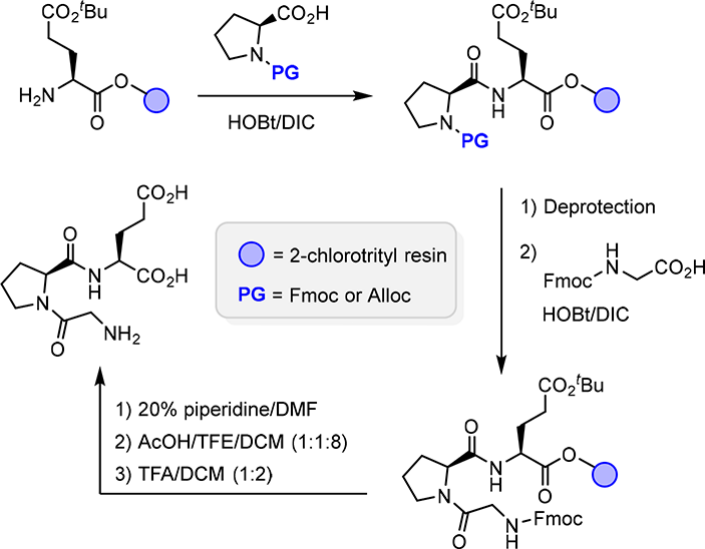
Representative SPPS for Glypromate (adapted^45^)

García-López’s group described an SPPS protocol for the synthesis of Glypromate analogs using a 2-chlorotrityl polystyrene resin.^45^ Following this methodology (Scheme 1), the resin is loaded with l-glutamic acid γ-*tert*-butyl ester.^45^ Then, using the chemistry of 1-hydroxybenzotriazole (HOBt) and *N*,*N*′-diisopropylcarbodiimide (DIC), central and N-terminal amino acids (N-protected as Fmoc or Alloc carbamates) are subsequently coupled.^45^ For the resin cleavage step, a mixture of acetic acid and 2,2,2-trifluoroethanol (TFE) followed by trifluoroacetic acid (TFA) is used, allowing the concomitant removal of the *tert*-butyl protecting group.^45^ Despite good yields (68–98%) and high purity (>95%),^45^ this methodology precludes the use of protected amino acids and, consequently, many deprotection steps. Furthermore, since this methodology requires that glutamate is covalently bonded to the resin through an ester bond with either the α- or γ-carboxylic acid, this protocol does not allow the use of bis-functionalized glutamates at the carboxylic acid moieties.

#### 2.3.2 LPPS-Based One-Pot Methodology

**Figure 3:**
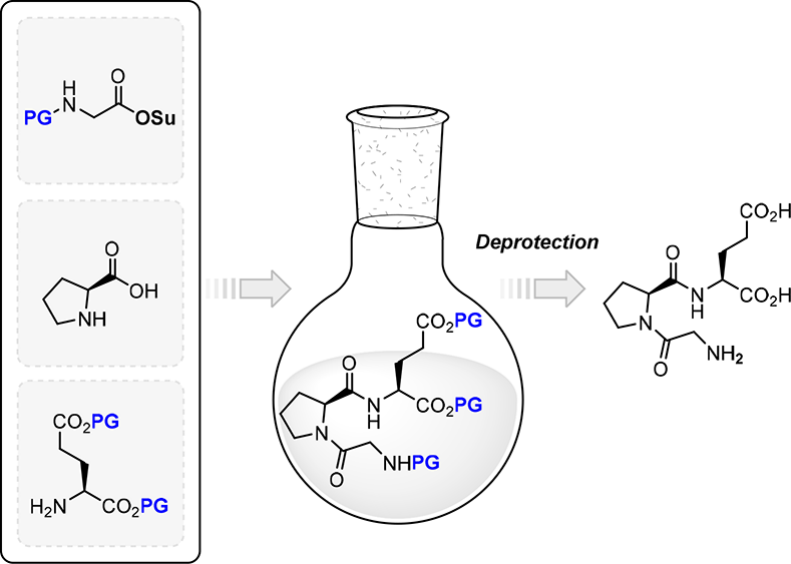
Representative one-pot synthesis for Glypromate (adapted^46^).

Sampaio-Dias and co-workers developed a chemoselective LPPS-based one-pot protocol (Figure 3) for the synthesis of oligopeptides without the need of isolating the intermediates, thereby reducing chemical waste and affording higher yields in comparison with classical approaches in LPPS.^46^

This protocol was subsequently adapted for the preparation of Glypromate and structure-related peptidomimetics following the principles of green chemistry.^47^ In this approach, hazard solvents (e.g., dichloromethane, dimethylformamide) are replaced by ethyl acetate (a more eco-friendly alternative). Following this protocol, proline reacts with preactivated glycine derivative [succinimidyl *N*-(benzyloxycarbonyl)glycinate] followed by *in situ* activation of the peptide intermediate with [(1*H*-benzotriazol-1-yl)oxy]tri(pyrrolidin-1-yl)phosphonium hexafluorophosphate (PyBOP). After the addition of glutamate α,γ-dibenzyl ester, the perbenzylated Glypromate derivative is obtained in 95% yield and 99% purity after precipitation.^48^ Glypromate is then obtained by hydrogenolysis catalyzed by Pd/C with concomitant removal of all protecting groups without the need for chromatography.^48^ Following this methodology, the overall process time is reduced for the synthesis of perbenzylated Glypromate (13–14 h) in contrast with classical protocols in LPPS (∼75 h). Furthermore, this protocol enables the preparation of structure-related analogs compatible with bis-functionalization of the glutamate residue.^48^ Green metrics demonstrate that this protocol is more eco-friendly based on EcoScale, a semiquantitative tool to evaluate the effectiveness of a synthetic reaction (EcoScale = 75),^48^ with the reduction of chemical waste as corroborated by the environmental factor (E-factor) obtained (E-factor = 1.7)^48^ in comparison with classical approaches (E-factor = 6.8).^49^

## 3 Glypromate Analogs

The development of Glypromate analogs is being explored to provide viable pharmaceuticals for the treatment of CNS-related disorders. In this sense, new analogs should display enhanced pharmacokinetic profiles such as improved BBB permeability, metabolic stability, and oral bioavailability.^49^

### 3.1 Modifications at the Glycine Residue

**Figure 4:**
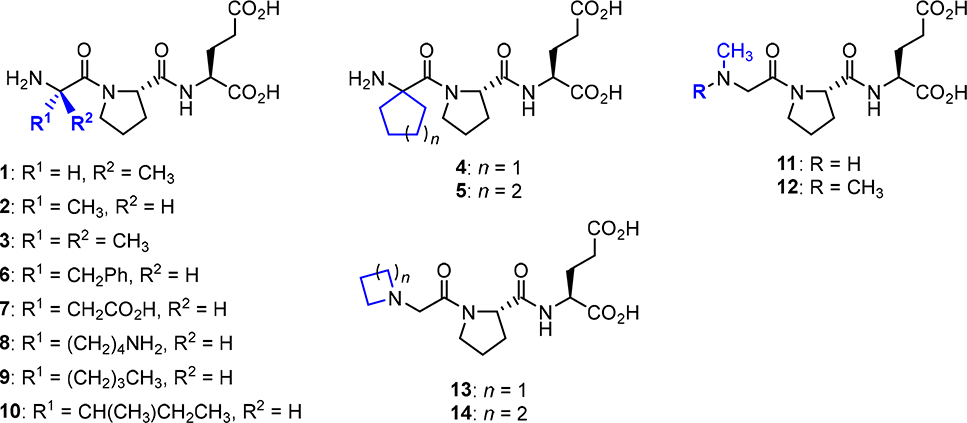
Glypromate modifications at glycine residue.^4,45,50,51,53^

Glycyl mimetics have been synthesized to explore the role of this amino acid in the neuroprotective profile of Glypromate and to improve activity.^4,45,50−52^ In 2005, Lai and co-workers reported the synthesis of several analogs modified at the glycine residue of Glypromate (Figure 4). In compounds **1** and **2** (Figure 4), glycine was replaced by d-alanine and l-alanine, respectively, while in analog **3**, the nonproteinogenic amino acid 2-methylalanine, also known as α-aminoisobutyric acid (Aib), was used instead.^4,50^ Other non-natural scaffolds such cyclopentyl (**4**) and cyclohexylglycine (**5**) were also used as glycine mimetics for the development of Glypromate analogs. Compounds **1**–**5** were able to enhance both lipophilicity and metabolic stability in comparison with the parent neuropeptide.^50^

García-López’s group studied the replacement of glycine with aromatic (Phe, **6**), acidic (Asp, **7**), basic (Lys, **8**), and aliphatic (Nle, **9**; Ile, **10**) amino acids.^45^ Most of the analogs synthesized demonstrated lower neuroprotective profiles in contrast with Glypromate. On the other hand, it was possible to observe an improvement in the displacement of [^3^H]-l-glutamate from rat brain synaptic membranes.^45,51^

Lai and co-workers also explored the introduction of diverse alkyl groups at the N-terminus of Glypromate (**11**–**14**). These derivatives were found to increase the lipophilicity in comparison with Glypromate, thus improving membrane permeability.^50^

In neuroprotective assays using striatal cells exposed to okadaic acid for 24 h, compound **2** demonstrated superior performance in comparison with Glypromate (recovery value of 26.4%, at 10 μM vs 20.1% for Glypromate, at 1 mM). Compound **12** has also been shown to have satisfactory results (recovery values of 30–35%, at 1 mM).

Considering the displacement of [^3^H]-l-glutamate, only compounds **1**–**3** and **6**–**10** were studied. Compounds **1**, **2**, and **6** demonstrated the highest binding affinities (*K*_i_ of 2.66 ± 0.31 μM, 5.40 ± 0.75 μM, and 4.85 ± 1.02 μM, respectively) in comparison with the parent peptide (*K*_i_ = 31.24 ± 15.65 μM in the same assay).^45^

### 3.2 Modifications at the Proline Residue

**Figure 5:**
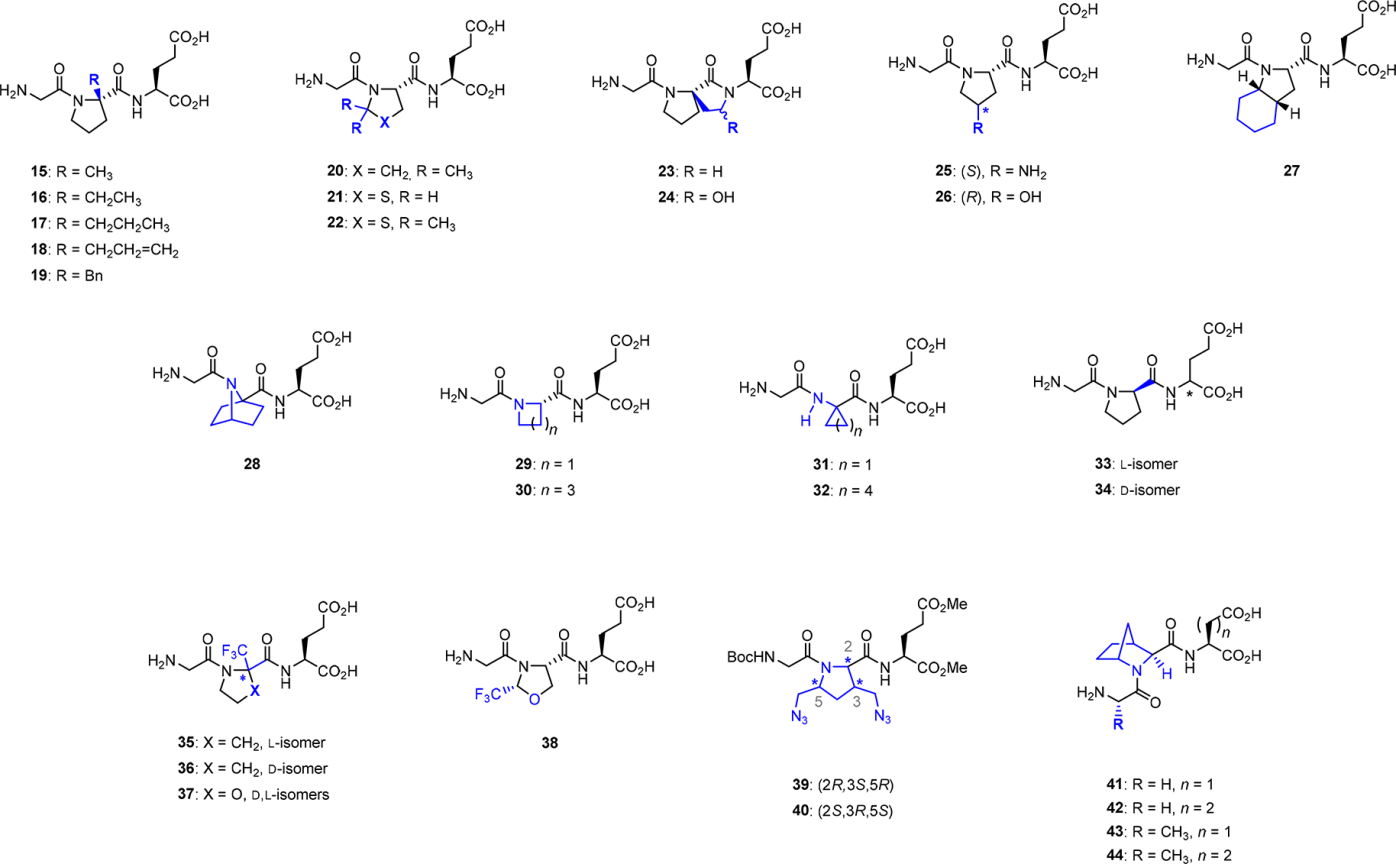
Prolyl-modified Glypromate analogs.^45,49,54,58−60^

Brimble’s group has synthesized several proline-modified Glypromate analogs (**15**–**24**, Figure 5), most of them exhibiting a preference for the *trans-*conformation and improved chemical and biochemical stability in comparison with the parent peptide.^54^ Compounds **15**–**19** (Figure 5) comprise a series of α-alkylated and benzylated analogs using Wang and Germanas’s modification of Seebach’s method of self-reproducing chirality.^54^ Among these compounds, α-methyl derivative **15** (also known as Trofinetide or NNZ-2566) displays a superior pharmacokinetic profile in comparison to that of Glypromate.^55,56^ Compound **15** displays improved half-life and oral bioavailability, enhanced protection against enzymatic degradation,^2^ and good anti-inflammatory and antiapoptotic properties.^57^*In vitro*, **15** significantly attenuated apoptosis in primary striatal cultures accounting for its neuroprotective effects,^55^ while *in vivo*, **15** reduced injury size in rats subjected to focal stroke.^55^ An intravenous infusion of **15** initiated 3 h after endothelin-induced middle-cerebral artery constriction and administrated for 4 h (3–10 mg kg^–1^ h^–1^), significantly reduced the infarcted area 5 days after administration.^55^ Neuroprotective efficacy in the middle cerebral artery occlusion mouse model was also observed following oral administration of the drug (30–60 mg kg^–1^) when formulated as a microemulsion.^55^ Recently, compound **15** (Trofinetide) has concluded phase III of clinical assays for the treatment of Rett syndrome. This a paradigmatic example of how a subtle chemical modification can convert a lead molecule into a pharmaceutical, and therefore this small peptide is explored in more detail in section 3.7.

Brimble’s group has also explored the incorporation of proline residues containing a pyrrolidine ring modified by replacing the γ-CH_2_ group with sulfur and/or incorporation of two methyl groups at C-5 (analogs **20**–**22**, Figure 5) and also analogs containing a spirolactam ring system (analogs **23** and **24**, Figure 5).^54^ Dimethylation at C-5 position of either pyrrolidine (**20**) or thiazolidine ring (**22**) was found to destabilize the *trans* conformation resulting in an increased population of the *cis* conformer,^54^ while unsubstituted thiazolidine ring (**21**) revealed the same *cis*–*trans* ratio found in Glypromate (*cis*:*trans*, 20:80).^49^ The data indicate that both compounds **15** (*trans* conformation) and **20** (*cis* conformation) display low binding affinities for the displacement of [^3^H]-l-glutamate from rat brain synaptic membranes (*K*_i_ = 7.96 ± 1.83 μM and *K*_i_ = 3.79 ± 0.53 μM) and are both able to prevent the death of hippocampal neurons caused by NMDA-induced excitotoxicity.^54,61^ Interestingly, the results obtained in glutamate receptors show that binding affinity does not correlate with *cis*–*trans* prolyl conformation. Moreover, despite compounds **21** and **22** not demonstrating a substantial affinity for glutamate receptor binding,^54,61^ compound **22** exhibited high protection against neuronal loss caused by NMDA excitotoxicity.^62^ Compounds **23** and **24** have in common a spirolactam bridge between the α-position of the proline and the nitrogen of the glutamate, which imprints conformational constraints with a preference for the *trans* counterpart^54^ and is detrimental to the bioactivity of these compounds.^24,54^

Using the SPPS strategy described in section 2.3.1, García-López’s group has developed a series of Glypromate analogs containing proline derivatives including constrained proline mimetics (compounds **25**–**32**, Figure 5). Binding affinity assays at glutamate receptors and neuroprotective assays in rat hippocampal neurons were performed for these analogs.^45^ Regarding the glutamate receptors binding affinity assays, the replacement of prolyl residue by *cis*-4-amino-l-proline (**25**), *trans*-4-hydroxy-l-proline (**26**), and (2*S*,3a*S*,7a*S*)-octahydro-1*H*-indole-2-carboxylic acid (**27**) displayed higher affinities (*K*_i_ = 15.54 ± 4.78, 9.24 ± 1.74, and 22.37 ± 5.30 μM, respectively) in comparison to Glypromate (*K*_i_ = 31.24 ± 15.65 μM).^45^ In contrast, when using l-azetidine-2-carboxylic acid (**29**), 1-aminocyclopropane-1-carboxylic acid (**31**), and 1-aminocyclohexane-1-carboxylic acid (**32**) as prolyl surrogates, these Glypromate analogs showed no binding affinities to glutamate receptors (*K*_i_ > 100 μM).^45^ In this series, compounds **28** (with b_7_Pro) and **30** (with a pipecolic acid residue) were found to exhibit the highest affinities toward glutamate receptors (*K*_i_ = 0.48 ± 0.09 and 2.39 ± 0.17 μM, respectively).^45^

Approximately half of the Glypromate neuroprotective activity toward NMDA excitotoxicity was retained by the conformationally restricted compounds **27**, **28**, and pipecolyl derivative **30**, while the best results were obtained for analogs **29**, **31**, and **32** (recovery between 27% and 34%, at 100 μM and *K*_i_ > 100 μM) in this study.^45^ However, none of these compounds were as effective as Glypromate (with 56% of recovery).^45^ These results might indicate that there is no correlation between [^3^H]-l-glutamate displacement and putative neuroprotective activity in rat hippocampal neurons when facing NMDA excitotoxicity.^24^

The effect of the d-stereochemistry at prolyl residue was also investigated by García-López’s group. Besides a d-proline, **33** and **34** (Figure 5) also display an l- or a d-glutamic acid residue, respectively. Both of these compounds exhibited good neuroprotection activity against NMDA excitotoxicity despite the lack of affinity for glutamate receptors.^24,61^

Simon and co-workers reported the synthesis of trifluoromethylated Glypromate analogs using α-trifluoromethylprolines (**35** and **36**) and trifluoromethyloxazolidine scaffolds (**37** and **38**) as proline surrogates (Figure 5).^58^ For the synthesis of **35** and **36** (Figure 5), the peptide coupling was applied from C- to N-terminus, while the reverse strategy was required for analogs **37** and **38** (Figure 5).^58^ Despite the successful preparation of these fluorinated Glypromate analogs and expected enhanced lipophilicity and chemical stability,^58,63^ biological evaluation was not performed.

Ferreira da Costa and co-workers have employed 3,5-bis(azidomethyl)pyrrolidines as proline surrogates to the synthesis of Glypromate analogs **39** and **40** (Figure 5).^59^ These functionalized pyrrolidine scaffolds were obtained as a racemic mixture from methyl 2-benzyl-2-azanorborn-5-ene-3-carboxylate (*exo* cycloadducts), as previously described in the literature.^64−66^ Following solution-phase peptide synthesis protocols, analogs **39** and **40** (Figure 5) were obtained as a mixture of diastereoisomers after fruitless efforts to separate them by chromatography.^59^ To date, no biological data have been reported for these compounds.

Sampaio-Dias and co-workers described the design and preparation of constrained analogs of Glypromate based on (1*R*,3*S*,4*S*)-2-azanorbornane-3-carboxylic acid (a hybrid construct of l-proline and l-pipecolic acid and also a structural isomer of b_7_Pro) as shown in Figure 5.^60^ These bicyclic Glypromate analogs (**41**–**44**, Figure 5) were successfully synthesized using the one-pot method previously described (section 2.3.2.).^46^ These compounds did not exhibit meaningful cytotoxicity in SH-SY5Y and human adipose mesenchymal stem cells up to 100 μM.^60^ Moreover, the neuroprotective activity of hybrid Glypromate analogs **41**–**44** was assessed in SH-SY5Y cells using 6-OHDA as a neurotoxic insult.^60^ All compounds of this series exhibited a significant (*p* < 0.01) increase in the recovery values after 6-OHDA injury, ranging between 24.7 and 40.0% at 100 μM, while the parent neuropeptide demonstrated a 12.8% recovery tendency (*p* = 0.0611) at the same concentration.^60^ Among this series, compound **41** exhibited the highest recovery value (40.0%, at 100 μM), being considered an interesting leading compound for further development.^60^

### 3.3 Modifications at the Glutamic Acid Residue

**Figure 6:**
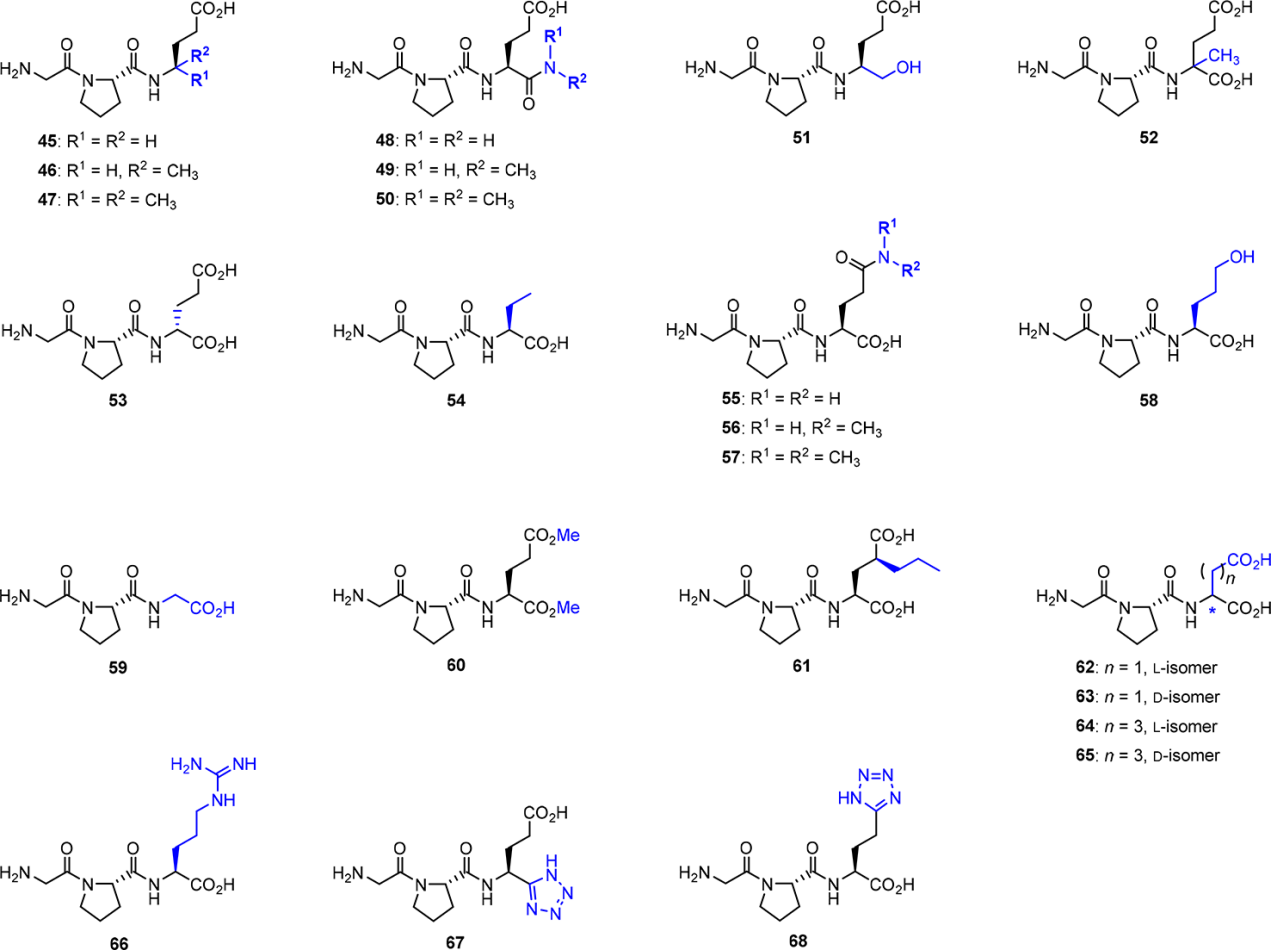
Glypromate modifications at glutamic acid residue.^49,61,67−70^

Brimble’s group has prepared several glutamate-modified Glypromate analogs (Figure 6).^49^ Compounds **45**–**53** were subjected to *in vitro* neuroprotective assays in striatal cells of Wistar rat embryos, and apoptosis was induced with okadaic acid. In this series, only compound **50**, which features a *N*,*N*-dimethylamide, showed neuroprotective activity between 1 and 100 μM with recovery values ranging from 20 to 40%,^49^ while the parent peptide displayed a similar effect (25–40%), albeit only at higher concentrations (1 mM).^49^ Lower percentages of recovery (20%) were obtained with 1 mM of analog **46**.^49^ None of the other compounds exhibited neuroprotective activity in this study.^49^

In another study, Brimble’s group explored the derivatization of the side chain of glutamic acid (compounds **54**–**61**, Figure 6) and performed neuroprotective assays in striatal cells obtained from Wistar rats embryos, in which apoptosis was induced with okadaic acid.^68^ Interestingly, the replacement of glutamate with a glycine residue in analog **59** exhibited neuroprotective activity with a recovery value of 20% at 1 mM, while the parent peptide displayed recovery values between 25 and 40% at the same concentration.^68^ Lower recovery values were obtained for the unsubstituted amide **55**, the alcohol **58**, and the dimethyl ester **60**, with recovery values ranging from 10 to 15% at 1 mM.^68^ None of the other analogs exhibited neuroprotective activity.^68^

García-López’s group has also developed a series of glutamate derivatives using stereoisomeric forms of aspartate (compounds **62** and **63**, Figure 6) and homoglutamic acid (compounds **64** and **65**, Figure 6).^61^ Displacement of [^3^H]-l-glutamate from rat brain synaptic membranes shows that these compounds display no affinity for glutamate receptors.^61^ In the same radiolabeled experiments, García-López’s group tested the ability to displace [^3^H]-l-glutamate binding at the NMDA receptors by compound **53** (Figure 6), which incorporates the d-isomer of glutamic acid.^61^ In contrast to its diastereoisomer, Glypromate, analog **53** did not displace it at concentrations up to 10^–4^ M.^61^ It is known that ligands interacting with the glutamate binding site of the NMDA receptors reveal the preference for the d-configuration, but some exceptions have been reported for both agonists and antagonists including d-glutamate which interact less potently with NMDA receptors than the l-isomer.^61,67,71^

Ioudina and Uemura explored compounds **62** and **66** (Figure 6), in which glutamic acid was replaced by l-aspartic acid and l-arginine residues, respectively, and evaluated their effect against Aβ-induced toxicity in cultured rat hippocampal neurons.^69^ Despite the lack of interesting results found for **62**, compound **66** (10–100 μM) was able to prevent an Aβ-mediated increase in lactate dehydrogenase (LDH) release and changes in the 3-(4,5-dimethylthiazol-2-yl)-2,5-diphenyltetrazolium bromide (MTT) reduction assay.^69^ For this reason, the potential antiapoptotic effect of this compound was studied. At 50 μM, compound **66** effectively prevented the Aβ-mediated activation of caspase-3 by Aβ_25–35_ (20 μM) and Aβ_1–40_ (5 μM).^69^ Similarly, **66** prevented an increase of p53-positive cells induced by Aβ peptides suggesting a neuroprotection mechanism based on the inhibition of the caspase-3/p53-dependent apoptosis.^69^ The authors claim that compound **66** has excellent antiapoptotic properties, and it may be a suitable candidate for the treatment of AD.^69^

Taking advantage of click chemistry, Brimble’s group synthesized Glypromate analogs using a tetrazole scaffold as a carboxylic acid surrogate at either the α (**67**) or γ (**68**) positions of the glutamic acid residue (Figure 6).^70^ Since the tetrazole moiety is often used to improve the metabolic stability and oral bioavailability of therapeutic agents, these analogs are expected to display enhanced pharmacokinetic profiles when compared to Glypromate, promoting their potential application in the treatment of traumatic brain injury.^70^ However, no biological data were provided.

### 3.4 Cyclic and Macrocyclic Analogs

The use of cyclic peptides is an elegant chemical approach for the preparation of constrained peptides with restricted geometries that can be used to probe their bioactive conformation.^72^ Macrocycles are cyclic structures involving at least 12 atoms.^73^ There are several possibilities for peptide macrocyclization to occur, such as head-to-tail, head-to-side-chain, side-chain-to-tail, or side-chain-to-side chain.^74^

**Figure 7:**
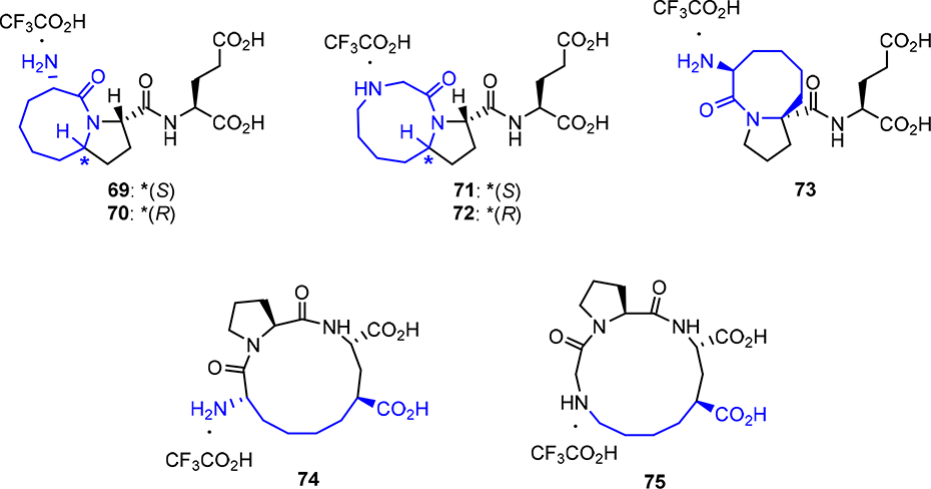
Cyclic and macrocyclic Glypromate analogs.^75−77^

Brimble’s group developed macrocyclic derivatives of Glypromate to constrain the conformation of the proline ring and provide the relationship between *cis*–*trans* conformers and bioactivity.^75,76^ Cyclic and macrocyclic derivatives **69**–**75** (Figure 7) adopt well-defined conformations around the Gly-Pro bond. Cyclic (**69**–**73**) analogs and macrocycle **74** (cyclotridecane) exhibit complete *trans* selectivity, while **75** (cyclotetradecane) displays a 65:35 (*trans*:*cis*) mixture of conformers.^76^ The lack of *cis*/*trans* selectivity of **75** is attributed to an increased flexibility of the amide bonds (Pro) embedded in the larger 14-membered ring in contrast with **74**.^76^ The exclusive detection of the *trans* conformer found in **74** may be attributed to the stabilization of the structure by the formation of a γ-turn and/or by being embedded in a smaller ring.^76^

Among cyclic derivatives **69**–**75**, it was found that **69** (100 pM, recovery value of 31.1%, *p* < 0.001) and **71** (100 pM, recovery value 30.6%, *p* < 0.001) demonstrate a superior neuroprotective profile compared with Glypromate (no numerical data presented) against 3-nitropropionic acid/glutamate in cerebellar microexplants.^77^ In addition, compound **71** (100 pM) has also been found to be neuroprotective against neuronal damage caused by HI injury induced by unilateral carotid artery ligation followed by inhalational asphyxia.^77^

These novel cyclic and macrocyclic Glypromate analogs were patented by Brimble’s group for application in the treatment or as a preventive therapy of CNS-related pathologies, albeit further studies need to be performed to fully disclose the therapeutical potential of these analogs.^77^

### 3.5 Pseudotripeptide Analogs

**Figure 8:**
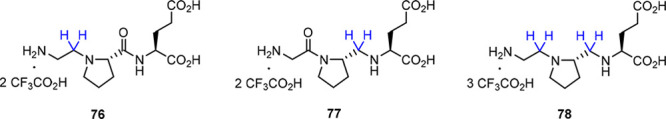
Amide-to-amine isosteres of Glypromate.^78^

Cacciatore’s group has explored pseudotripeptides of Glypromate through the introduction of amide bond isosteres (aminomethylene units) to increase metabolic stability.^78^ In this work, one (Gly-Pro or Pro-Glu, **76** and **77**, respectively, Figure 8) or both (**78**, Figure 8) amide bonds were replaced by aminomethylene groups. Pseudopeptides **76**–**78** exhibit good water solubility and stability in human plasma.^78^ While Glypromate exhibits a half-life of only 30 min in human plasma, all of these analogs exhibited improved half-life profiles.^78^ In this series, compound **78** (*t*_1/2_ = 11.8 h) demonstrated the highest half-life in plasma, followed by **76** (*t*_1/2_ = 6.6 h) and **77** (*t*_1/2_ = 4.5 h).^78^

The potential antineuroinflammatory effects of amide isosteres **76**–**78** (10 μM) were studied in Aβ_25–35_, phorbol 12-myristate 13-acetate (PMA), or lipopolysaccharide (LPS)-treated THP-1 cells, a transformed human monocyte cell line used as a model for microglia.^78^ In this assay, the interleukin-1β (IL-1β), interleukin-18 (IL-18), monocyte chemoattractant protein 1 (MCP-1), and tumor necrosis factor-α (TNF-α) expression were analyzed. Interestingly, these three pseudotripeptides (**76**–**78**) differentially modulated cytokine production in PMA, LPS, or Aβ_25–35_-activated THP-1 cells.^78^ In PMA-stimulated cells, isostere **76** denoted a proinflammatory effect by increasing the levels of IL-1β (*p* < 0.05) and anti-inflammatory effects by reduction of MCP-1 expression.^78^ Compounds **77** and **78** denoted a statistical reduction (*p* < 0.05) of MCP-1 and TNF-α expression, while **77** also reduced the levels of IL-18 and **78** decreased the IL-1β expression.^78^ In this experiment, Glypromate only exhibited a statistical (*p* < 0.05) reduction of IL-18 and MCP-1 expression.^78^ In Aβ_25–35_-treated THP-1 cells, only **78** exhibited anti-inflammatory activity with a statistical decrease (*p* < 0.05) of TNF-α.^78^ In contrast, **76** and **77** exhibited proinflammatory responses (*p* < 0.05) by increasing the expression of IL-18, and in the case of **76**, IL-1β expression was also exacerbated.^78^ In this experiment, Glypromate did not induce a significative alteration in the expression of the cytokines under study.^78^ In LPS-treated THP-1 cells, **76** and **77** statistically (*p* < 0.05) induced a reduction of TNF-α expression, while **78** exhibited a proinflammatory response (*p* < 0.05) by increasing the IL-1β levels.^78^ Glypromate was devoid of any significant effects on cytokines expression.^78^

In the same study, Cacciatore’s group evaluated the potential protective effect of **76**–**78** (10 μM) in THP-1 cells treated with Aβ_25–35_ (10 μM) or H_2_O_2_ (300 μM) as the toxic stimuli.^78^ After a 24-h of incubation with Aβ_25–35_ or H_2_O_2_, cell viability decreased by 40% and 43%, respectively, with respect to the control (MTT reduction assay).^78^ In Aβ_25–35_-treated THP-1 cells, compound **78** promoted a statistical increase in the survival rate by 76% (*p* < 0.05).^78^ In H_2_O_2_-treated cells, all compounds (**76**–**78**) were able to significantly counteract the cytotoxicity observed, with emphasis on **76** and **77**, which promoted the increase in the survival rates in 90 and 100% (*p* < 0.05), respectively.^78^ In both groups, Glypromate exhibited high cytoprotection (>90%, *p* < 0.05).^78^

In follow-up work, Cacciatore’s group further explored pseudotripeptides **76**–**78** as potential drug candidates to tackle AD.^79^ First, neuroprotection was evaluated using differentiated SH-SY5Y cells and cell viability was determined by the MTT reduction assay or the LDH assay. In the MTT reduction assay, Glypromate and compounds **76**–**78** (100 μM) inhibited cell death induced by Aβ_1–42_ exposure (20 μM), with a significant increase (*p* < 0.05) in the cell viability by 13.1, 31.8, 12.0, and 16.4%, respectively.^79^ In the LDH assay, Glypromate and compounds **76**–**78** (100 μM) protected SH-SY5Y cells from Aβ_1–42_-induced membrane damage, resulting in a significant increase (*p* < 0.05) in the cell viability by 20.5, 41.7, 19.5 and 20.3%, respectively.^79^ The neuroprotective effect obtained for Glypromate and analogs **76**–**78** was comparable to that of memantine, which was used as a positive control of clinical relevance.^79^

The effect on acetylcholinesterase (AChE) activity was also evaluated. In fact, Glypromate and compounds **76**–**78** (at 100 μM) induced a statistical reduction (*p* < 0.05) of Aβ_1–42_-stimulated (20 μM) AChE activity at a rate of 19.35, 20.96, 16.93, and 18.54%, respectively.^79^ Although positive, this effect is only slim as GAL (galantamine hydrobromide, an anticholinergic drug) managed to significantly decrease AChE activity at a much lower concentration of 0.1 μM. It is known that Aβ_1–42_ affects secretase activity. As such, the activity of α- and β-secretase was evaluated. Glypromate and compound **76** (at 50 and 100 μM) managed to increase α-secretase activity against Aβ_1–42_-induced inactivation.^79^ However, different concentrations of Glypromate and compounds **76**–**78** did not modify β-secretase activity upon Aβ_1–42_-induced stimulation in differentiated SH-SY5Y cells.^79^

In this study, it was also found that Glypromate and compounds **76**–**78** (100 μM) promoted 2.37-, 2.51-, 2.16-, and 1.97-fold changes, respectively (*p* < 0.05), in the total antioxidant capacity (TAC) levels.^79^ Although no significative differences (*p* > 0.05) were found in the total oxidative status (TOS) levels in the presence of these compounds when compared to the untreated controls, compound **76** was able to alleviate oxidative stress in a concentration-dependent manner (1–100 μM) upon Aβ_1–42_ exposure.^79^ Using Hoechst 33258 staining, it was possible to access the morphology of the apoptotic cells. After flow cytometry analysis, compound **76** demonstrated to be the most effective against Aβ_1–42_-induced apoptosis (*p* < 0.05), followed by Glypromate, **77**, and **78** (all at 50 μM).^79^ Since the analog with better results was compound **76**, the authors decided to apply RT^2^ Profiler PCR arrays to determine the exact molecular mechanism of its inherent neuroprotection. After treatment with **76** (50 μM) no significant expressional alteration on apoptosis or necrosis-related genes was observed.^79^ However, this compound was able to modulate the Aβ_1–42_-induced alteration of *BRCA1*, *AKT1*, *BCL2*, *BCL2L*, *CASP8*, *CASP9*, and *FASLG* gene expression.^79^

Considering these results, the most promising analog is **76** but pseudopeptides **77** and **78** are also great candidates to counteract neuroinflammation processes in AD.^78,79^

### 3.6 Glypromate Conjugates

#### 3.6.1 PEGylated Glypromate

**Figure 9:**
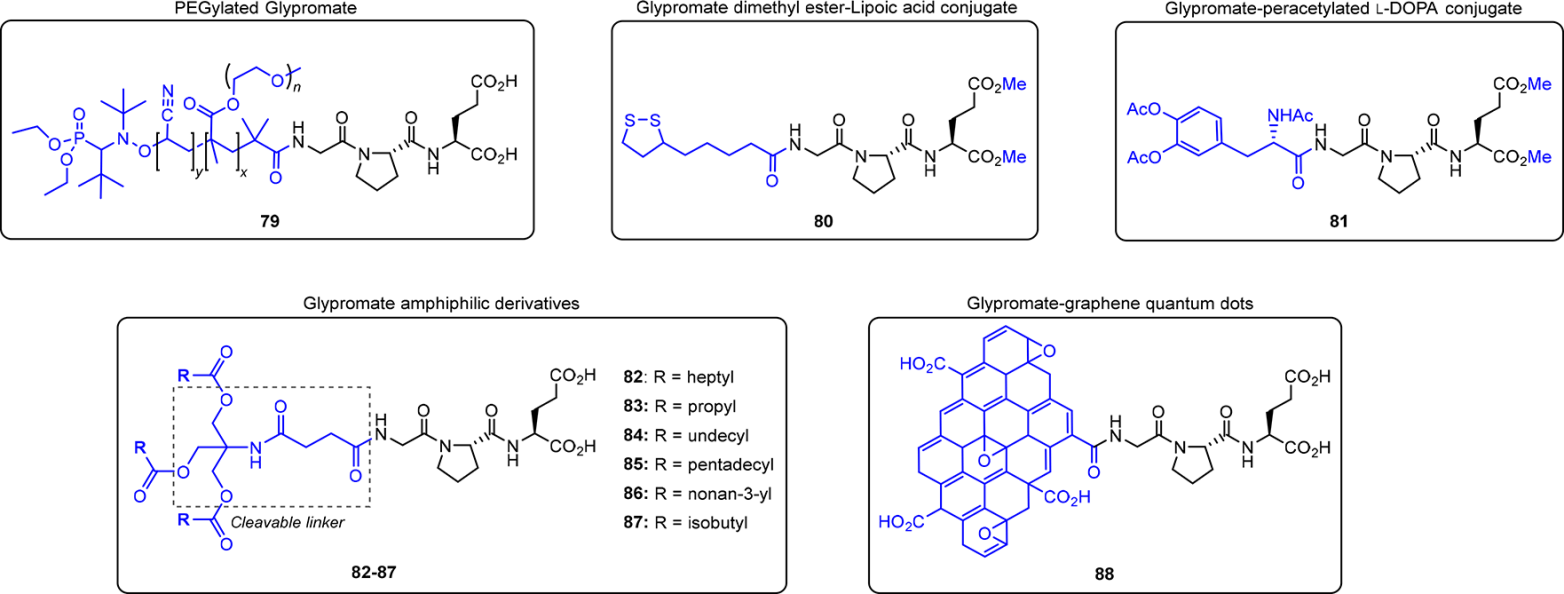
N-terminal conjugation of Glypromate with different carboxylic acids.^19,43,80−84^

Nicolas’ group reported the conjugation of Glypromate with polyethylene glycol (PEG) (**79**, Figure 9) using a new technique of PEGylation by nitroxide-mediated polymerization system based on α-functional comb-shaped polymethacrylates with PEG side chains.^19^ The synthetic strategy relies on the polymerization of poly(ethylene glycol) methyl ether methacrylate (MePEGMA) initiated by SG1-based alkoxyamines bearing an *N*-hydroxysuccinimidyl (NHS) moiety,^19^ affording PEGylated Glypromate in nearly quantitative yield.^19^ The authors advance that using a copolymer of MePEGMA with acrylonitrile, PEGylated Glypromate micelles or nanoparticles could be synthesized to permeate the BBB and also extend the plasma half-life of Glypromate.^19^ Despite the potential applications, no biological data were provided by the authors in this regard.

#### 3.6.2 Glypromate Dimethyl Ester–Lipoic Acid Conjugate

In an effort to develop multifunctional agents with neuroprotective activity, Cacciatore’s group synthesized a Glypromate conjugate (**80**, Figure 9) with lipoic acid (LA).^80,85^ Conjugate **80** was synthesized by amide bond formation between the carboxylic acid of LA and the N-terminal of the dimethyl ester Glypromate (analog **60,**Figure 6).^80^

The partition coefficient (log *P*) value obtained (log *P* = 1.51 ± 0.02) indicates that this conjugate might display good intestinal absorption.^80^ Furthermore, conjugate **80** displays adequate water solubility (8.65 ± 0.35 mg mL^–1^), which is significantly lower in an acidic medium (pH 1.3 solution, 2.45 ± 0.09 mg mL^–1^), in contrast with physiologic pH (pH 7.4 solution, 8.17 ± 0.23 mg mL^–1^).^80^ Chemical stability studies were performed on pH = 1.3 and 7.4, providing evidence that conjugate **80** improves the chemical stability in both conditions (*t*_1/2_ = 58.75 ± 1.76 h at pH = 1.3 and *t*_1/2_ = 217.17 ± 2.35 h at pH = 7.4), in comparison with the parent peptide (*t*_1/2_ = 51.73 ± 2.1 h at pH = 1.3 and *t*_1/2_ = 151.23 ± 2.12 at pH = 7.4).^80^

The plasma stability of conjugate **80** was also studied. Using 80% rat plasma in PBS (pH = 7.4), conjugate **80** exhibited a slight decrease in half-life (*t*_1/2_ = 0.085 ± 0.004 h) in comparison to that of Glypromate (*t*_1/2_ = 0.108 ± 0.004 h). In 80% human plasma, **80** exhibited an extended half-life (*t*_1/2_ = 3.14 ± 0.08 h), which represents a 9-fold increase of stability when compared to Glypromate under the same conditions (*t*_1/2_ = 0.35 ± 0.02 h).^80^

Using a parallel artificial membrane permeability assay model of BBB (PAMPA-BBB), the permeability of **80** was determined at pH = 7.4 (18 h), whereas for accurate prediction of oral absorption, the permeability in the PAMPA assay was conducted at pH = 5.0, 6.5, and 7.4 to mimic the physiological conditions of the gastrointestinal tract.^80^ Conjugate **80** showed a good pH-dependent permeability profile through the gastrointestinal PAMPA membrane assay (*P*_e_ = 6.45 × 10^–6^ cm s^–1^), and it was classified as a compound with high CNS permeability.^80^

Using both undifferentiated and differentiated neuroblastoma SH-SY5Y cells, cytotoxicity and neuroprotection assays were performed for conjugate **80** and compared to LA and Glypromate.^80^ The cytotoxicity of LA, Glypromate, LA + Glypromate (equimolar mixture), and **80** was studied at 1, 10, 100, 300, and 500 μM using the MTT reduction assay. In this assay, conjugate **80** displayed the lowest cytotoxicity, followed by Glypromate, LA, and LA + Glypromate conditions (no numerical data provided).^80^ All the tested compounds only exhibited significant cytotoxic effects at the highest concentrations tested (300 and 500 μM).^80^ For that reason, the concentration of **80** in the neuroprotection studies was set as 100 μM (the highest concentration without noticeable cytotoxicity), in the presence of H_2_O_2_ (25, 150, and 300 μM) and 6-OHDA (50, 75, and 150 μM) as the neurotoxic insults.^80^ Here, the same trend was also observed in the neuroprotection assays (MTT reduction assay), with **80** displaying the best neuroprotective performance in comparison with Glypromate and LA alone or their equimolar mixture.^80^

In follow-up work, Cacciatore’s group studied the *in vitro* neuroprotective effects of conjugate **80** and Glypromate in an AD model using differentiated human neuroblastoma SH-SY5Y cells in the presence of Aβ_1–42_ using the MTT reduction assay and the LDH release assay.^43^ Furthermore, AChE activity, TAC, TOS levels, and neural cell apoptosis and necrosis were also evaluated. In addition, the biological safety of these novel formulations was evaluated in human blood cells using different cytotoxicity and genotoxicity assays.^43^ Both Glypromate and conjugate **80** (0.1–100 μM) were able to provide significant (*p* < 0.01) prevention toward neuronal cell death when assessed by MTT and LDH assays after Aβ_1–42_ exposure.^43^ This conjugate also reduced Aβ-induced AChE activity by 6.9% at 25 μM and 11.8% at 50 μM, demonstrating a slight improvement in comparison with the parent neuropeptide (Glypromate reduced Aβ-induced AChE activity by 5.46% at 25 μM and 10.36% at 50 μM).^43^ However, no statistical analysis was given for these results. In the TAC and TOS assays, both compounds (at 25 and 50 μM) co-treated with Aβ_1–42_ were able to successfully increase TAC and reduce TOS *in vitro* in comparison with the control (Aβ_1–42_-treated cells), with Glypromate exhibiting slightly better performance than conjugate **80**.^43^ Apoptosis detection employing Hoechst 33258 staining demonstrated that conjugate **80** inhibited apoptosis induced by Aβ_1–42_. The apoptosis–necrosis assay revealed that this conjugate protected from Aβ_1–42_-induced necrosis in a dose-dependent manner.^43^ Moreover, biosafety assays in cultured peripheral human whole blood cells demonstrated that **80** (0.1–100 μM) did not cause significant changes in cell viability after a 24-h incubation time (determined by MTT and LDH assays).^43^ As no significant changes were verified for the frequencies of sister chromatid exchange with increasing concentrations of Glypromate and conjugate **80** in cultured human lymphocytes, it can be assumed that they do not have genotoxic potential.^43^

#### 3.6.3 Glypromate–Peracetylated L/D configuration-DOPA Conjugate

Following the same strategy previously reported for the preparation of **80** (Figure 9), Cacciatore’s group described the conjugation of peracetylated levodopa (L/D configuration-DOPA) to the N-terminal of compound **60** via an amide bond (conjugate **81**, Figure 9).^81^

C57BL/6 mice, after chronic administration of low doses of 1-methyl-4-phenyl-1,2,3,6-tetrahydropyridine (MPTP), were used as an animal model of progressive chronic PD. Conjugate **81** increased the protection of TH-containing neurons (89 ± 8% viability compared to 78 ± 9% of L/D configuration-DOPA) against MPTP treatment (55 ± 7%), nearly restoring the resting morphology and reducing oxidative stress.^81^ In fact, the authors found that the administration of conjugate **81** suppressed the inflammatory nuclear factor κB (NF-κB) signaling and led to strong activation of the antioxidant-related nuclear factor erythroid 2–related factor 2, with striatum glutathione (GSH) levels and heme oxygenase 1 (HO-1) expression/protein levels restored/increased.^81^ Moreover, in animals treated with conjugate **81**, a neuroprotective effect was observed by the reduction of glial reactivity,^81^ demonstrating better therapeutic properties when compared with L/D configuration-DOPA alone, holding promise for new therapeutical strategies. Therefore, conjugate **81** has an interesting profile toward PD, as it changes features related to the pathophysiology of the disease, namely, inflammation and oxidative stress.

#### 3.6.4 Glypromate Amphiphilic Derivatives

Brimble’s group reported the preparation of Glypromate amphiphilic derivative **82** (Figure 9) by a modular approach. For this purpose, Glypromate was used as the functional polar head, succinyl-Tris (Tris, 2-amino-2-hydroxymethylpropane-1,3-diol) as a cleavable linker, and three octyl chains attached to the linker as the hydrophobic tail (**82**, Figure 9).^83^

The self-assembled analog **82** displays long-range order forming a lamellar phase.^83^ Using Pluronic F-127, a nonionic copolymer surfactant, **82** can be dispersed as nanometer-sized colloidal stable particles.^83^*In vitro* experiments using human microvascular cell line 1 showed that Glypromate and the amphiphilic analog **82** (in the absence of Pluronic F-127) were able to inhibit cell growth with a similar half-maximal inhibitory concentration.^83^ However, the choice of this cell-based assay was not justified and the relevance of such experiments was not discussed. Moreover, no data were provided on this amphiphilic derivative in the context of neuroscience research.

In follow-up work, Brimble’s group further expanded the series of amphiphilic Glypromate analogs (**83**–**87**, Figure 9) using an improved SPPS protocol.^82^ In this approach, several fatty acids were used as the hydrophobic tail, keeping the succinyl-Tris motif as the linker.^82^ Despite the successful preparation of amphiphilic analogs **82**–**87**,^82,83^ so far no biological data on permeability and neuroprotective activity have been reported to support the applicability of these drug delivery formulations. However, the increased resistance of Glypromate-based nanoparticles against hydrolytic and proteolytic degradation is expected to improve the *in vivo* half-life of Glypromate and may allow the sustained release of Glypromate in the bloodstream or the CNS.^82^

#### 3.6.5 Glypromate–Graphene Quantum Dots

Liu’s group has designed a new nanomaterial based on the chemical conjugation of graphene quantum dots (GQD) with Glypromate neuropeptide (**88**, Figure 9),^86^ taking advantage of this carbon-based nanomaterial with low toxicity, low cost, large specific surface area, good solubility, and small size (being expected to cross the BBB).^86−89^ Additionally, GQD are known to inhibit the aggregation of Aβ_1–42_ and rescue the cytotoxicity promoted by Aβ oligomers.^86^ Conjugate **88** was prepared using 1-[3-(dimethylamino)propyl]-3-ethylcarbodiimide (EDC) and NHS.^84,86^

*In vitro* assays to study the effects of GQD and conjugate **88** in the aggregation of Aβ_1–42_ using the Thioflavin-T (ThT) fluorescence method were performed.^84^ In this assay, ThT is able to associate rapidly with aggregated Aβ_1–42_ fibrils but not with monomers or dimers of Aβ_1–42_.^84^ Both GQD and conjugate **88** exhibited better inhibitory effects on Aβ_1–42_ aggregates than resveratrol (positive control and reference drug) at the concentration of 200 μg mL^–1^.^84^ These results were further substantiated by transmission electronic microscopy images, in which only a few short linear Aβ_1–42_ fibrils or few amounts of amorphous aggregates were observed, being also confirmed by circular dichroism analysis.^84^ Hemolysis assays showed that conjugate **88** was found to display good hemocompatibility up to 500 μg mL^–1^ (hemolysis: 0.13, 0.18, and 0.29%, at 50, 200, and 500 μg mL^–1^, respectively).^84^ Lastly, **88** has a larger contact area than GQD, increasing its inhibitory capacity of Aβ_1–42_ aggregation.^84^

To further investigate the mechanisms underlying the efficacy of conjugate **88** in the pathogenic processes of AD, biocompatibility, behavioral, and biochemical studies were performed in SPF 6-month-old male APPswe/PS1dE9 double transgenic mice (APP/PS1) to which an intravenous injection with 20 mg kg^–1^ of **88** (200 mg mL^–1^) was given for 14 days.^84^ No histopathological abnormalities were detected in the liver or kidney.^84^ In the Morris water maze, conjugate **88** improved the spatial learning impairment in APP/PS1 transgenic mice and related forms of learning and memory in comparison with untreated and wild-type groups.^84^ Quantification of Aβ_1–42_ and Aβ_1–40_ in brain tissue and serum by enzyme-linked immunosorbent assay demonstrates that **88** can significantly decrease the amount of Aβ in both brain and serum of the APP/PS1 transgenic mice,^84^ which is corroborated by the reduced surface area of Aβ plaque by immunohistochemical staining in comparison with untreated mice.^84^ By using immunohistochemical techniques, conjugate **88** has also been shown to reduce microglial activation (compared to the untreated group) by reducing the Aβ aggregation.^84^ When assessing the impact of **88** on inflammation in APP/PS1 (suspension array with cytokines and chemokines), the results showed that, in the presence of **88**, the proinflammatory factors (e.g., IL-1α, IL-1β, IL-6, IL-33, IL-17α, TNF-α, and MIP-1β) decreased and the anti-inflammatory factors (IL-4 and IL-10) increased (compared with the untreated group).^84^

Still in the mentioned study and to assess the neuroprotective properties of **88**, levels of nerve growth factor (NGF) and brain-derived neurotrophic factor (BDNF) in the APP/PS1 mice brain were determined since NGF is essential for the survival of memory-related neurons^90^ and BDNF regulates synaptic plasticity.^91^ In APP/PS1 transgenic mice treated with conjugate **88**, levels of both NGF and BDNF were significantly increased (*p* < 0.01), which the authors attribute to the reduction of Aβ levels.^84^ Additionally, by performing the diolistic labeling method on hippocampal slices, the authors have shown that conjugate **88** promoted an increase in the number of dendritic spines in comparison with the untreated group, which may indicate that this conjugate is able to induce synapsis protection effects, possibly by the reduction of Aβ aggregation.^84^

Conjugate **88** was also found to promote significant neurogenesis in APP/PS1 mice since the number of newborn neural progenitor cells (NPC) and neurons in the hippocampus increased when compared to the untreated group, which can impact the cognitive deficit in AD.^84^

Despite the great results observed for conjugate **88**, no data were provided concerning chemical characterization and the reproducibility of the synthesis protocol. The intrinsic heterogeneity of these nanomaterials makes comparisons of properties uninformative. Therefore, the applicability of this conjugate is debatable. Moreover, Glypromate and GQD controls were not included in the neuroprotection and behavioral studies. Furthermore, although authors attribute these results to the action of the conjugate **88** in decreasing Aβ aggregation, no data on the effect of BDNF, NPC, and NGF levels in a nontransgenic animal model were provided as control.

#### 3.6.6 Glypromate–Bioactive Amines

Silva-Reis and co-workers have designed Glypromate conjugates with several active pharmaceutical ingredients (API) used in the chemotherapy of PD (amantadine) and AD (memantine) and also with (*R*)-1-aminoindane, a neuroprotective metabolite obtained from rasagiline (used as monoamine oxidase-B inhibitor in Parkinson’s therapy) to explore possible synergistic effects.^92^ Considering that Glypromate metabolism occurs from C- to N-terminal,^8^ these API were coupled at glutamate residue via an amide bond (at either the α- or γ-carboxylic acid).^92^ As such, this conjugation strategy is likely to improve the resistance toward enzymatic degradation and/or promote the sustained release of both API and Glypromate.^92^

**Figure 10:**
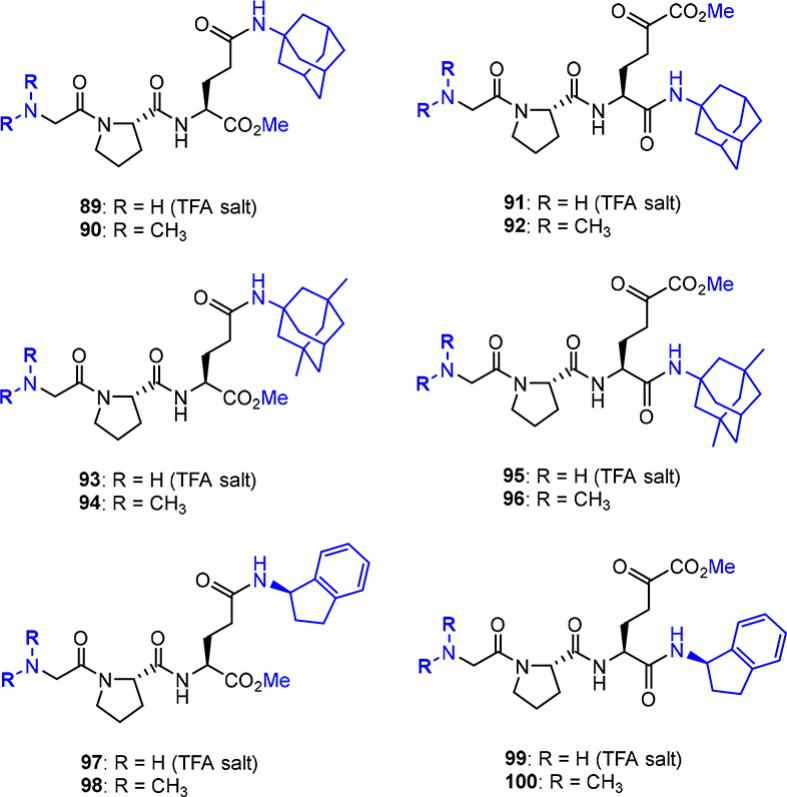
C-terminal conjugation of Glypromate with bioactive amines.^92^

A series of 12 conjugates (**89**–**100**, Figure 10) were synthesized using the one-pot synthesis methodology in solution-phase previously described (section 2.3.2.).^46,92^ Capping strategies of polar exposed groups were envisioned to increase the lipophilicity of the conjugates, such as N,N-dimethylation of the glycine residue (such as **12**, Figure 4) and esterification of the nonfunctionalized carboxylic acid group of the glutamate residue (such as **60**, Figure 6).^92^

Some physicochemical properties of the conjugates were calculated to predict bioavailability and BBB permeability. Considering the clogP values obtained, all the conjugates exhibited higher lipophilicity (clogP ranging from −1.00 to 1.73) in comparison to that of Glypromate (clogP = −4.39). As expected, the conjugates containing the *N*,*N*-dimethylglycine residue (conjugates **90**, **92**, **94**, **96**, **98**, and **100**) displayed the highest clogP; as so, these compounds are expected to exhibit improved pharmacokinetic profiles.^92^*In silico* BBB permeability studies show that these conjugates (except for **93**, **94**, **97**, and **98**) showed superior BB ratios (BB ratio up to 0.739) compared with Glypromate (BB ratio = 0.517) and therefore are likely to display better distribution than the parent neuropeptide.^92^ To date, no biological data were provided.

### 3.7 Trofinetide, a Successful Prolyl-Constrained Analog of Glypromate

Trofinetide (**15**, Figure 5) was developed by Brimble’s group and is currently being explored by Neuren Pharmaceuticals Ltd. and Acadia Pharmaceuticals Inc. in several neurological conditions.

Two double-blind clinical studies (phase II) performed in adults and pediatric patients demonstrated clinically promising results with significance over the placebo group for the treatment of the core Rett syndrome symptoms.^93,94^ For the trials with adolescent and adult females (56 females, aged between 15.9 and 44.2 years old), doses of 35 mg kg^–1^ or 70 mg kg^–1^ were used twice daily over 28 days and were well tolerated, with the highest doses exhibiting significant (*p* < 0.2) improvement relative to the control groups.^93^ In this exploratory study, the prespecified use of *p* < 0.2 was used as the criterion for the assessment of benefit across multiple end points due to the limited sample size.^93^ As so, Trofinetide was associated with a benefit over placebo (*p* < 0.2) in three core variables from three different efficacy domains: motor behavior assessment scale (*p* = 0.146), indicating improvement in major signs and symptoms of Rett syndrome;^93^ clinical global impression-improvement (CGI-I) score (*p* = 0.164), denoting an improvement in illness compared to baseline;^93^ and caregiver top 3 concerns visual analog scale score (*p* = 0.076), indicating improvement in the symptomatology as identified by caregivers.^93^

As for the pediatric study (girls aged between 5 and 15 years old), doses of 50, 100, or 200 mg kg^–1^ were tested twice daily over 42 days, with the highest doses exhibiting significant (*p* < 0.05) improvement relative to the control groups in three out of five core measures: Rett syndrome behavior questionnaire (RSBQ, total score, core neurobehavioral symptoms, *p* = 0.042), CGI-I (overall clinical status, *p* = 0.029), and Rett syndrome-clinician domain specific concerns (concerning aspects identified by clinicians, *p* = 0.025).^94^ Oral administration of Trofinetide appears to be a safe procedure, as only mild adverse effects were observed (e.g., gastroesophageal reflux).^93,94^ The pharmacokinetic parameters were consistent among the groups tested and indicate an elimination half-life of around 5–6 h, with body weight having a significant impact on the clearance and distribution volume of Trofinetide.^94^ Moreover, differences in bioavailability were evident between doses taken in different day periods (morning and evening), which might be due to variations in the metabolism or gastrointestinal absorption due to food intake.^93,94^

As of July 2022, Acadia Pharmaceuticals Inc. has submitted a new drug application to the U.S. Food and Drug Administration (FDA) for Trofinetide, in partnership with Neuren Pharmaceutical Ltd., for the treatment of Rett syndrome in adults and pediatric patients (two years of age and older) after statistically significant and clinically meaningful results over placebo from a phase III trial (Lavender study^95^). Lavender study was a 12-week, randomized, double-blind, placebo-controlled clinical trial to assess the effectiveness and safety of Trofinetide in 187 girls and young women with Rett syndrome.^95^ Both a caregiver evaluation (RSBQ) and a physician assessment (CGI-I) were included as coprimary outcomes in this study.^95^ The communication and symbolic behavior scales developmental profile infant–toddler checklist–social composite score (CSBS-DP-IT-Social), which measures communication skills, was the secondary end point for the caregiver evaluation.^95^

On the co-primary objectives, the Lavender study showed a statistically significant improvement compared to the placebo.^96^ The CGI-I scale score changed at 12 weeks (*p* = 0.0030; impact size = 0.47) and both RSBQ and CSBS-DP-IT-Social changed from baseline to 12 weeks (*p* = 0.0175 and effect size = 0.37; *p* = 0.0064 and effect size = 0.43, respectively).^96^ In the U.S., Trofinetide has been given orphan drug designation (ODD) and fast track status for the treatment of Rett syndrome, thus being eligible for priority review.^96^ The FDA has granted Trofinetide the rare pediatric disease designation,^96^ which is attributed to drugs under development for rare childhood diseases. As of September 13, 2022, Acadia Pharmaceuticals announced that the FDA granted a priority review for Trofinetide and set March 12, 2023, as the Prescription Drug User Fee Act date.^97^

Currently, Neuren has also conducted a phase II of clinical trials with Trofientide for fragile X syndrome which has shown clinical improvement in many of the core symptoms^98,99^ and has received ODD and fast track status for the treatment of fragile X syndrome by the FDA.^98^ Glass and co-workers also patented the use of Trofinetide for the treatment of autism spectrum disorders denoting its effectiveness in managing neurodegeneration, promoting neuronal function, and treating seizure activity and other symptoms related to these disorders.^100−102^

The replacement of the α-proton of proline by a methyl group in Glypromate neuropeptide is known to cause significative metabolic resistance toward protease activity, thereby extending the half-life of this peptidomimetic in rat plasma (up to 20 min).^55,103^*In vivo* studies performed by Bickerdike and co-workers demonstrated that Trofinetide has a blood half-life of 49 min (3 male and 3 female Sprague-Dawley rats) after intravenous administration.^55^ Moreover, the half-life in brain extracellular fluid (after 3 h infusion) was 74 min (5 male Sprague-Dawley rats).^55^ The same authors reported that the administration of Trofinetide (30 mg kg^–1^) by oral gavage denoted enhanced bioavailability (faster appearance in the blood and higher AUC_0–4 h_) in microemulsion formulation (5.15 μg h mL^–1^) when compared to saline formulation (2.85 μg h mL^–1^).^55^

Guan and co-workers also reported that Trofinetide has improved enzymatic stability with a prolonged plasma half-life and high neuroprotective capacity after ischemic brain injury in both adult and neonatal rats.^38^*In vitro* studies demonstrated that this analog significantly attenuates apoptotic cell death in primary striatal cultures. Furthermore, *in vivo* assays showed the reduction of injury size in rats subjected to focal stroke^104^ and the attenuation of ballistic type traumatic brain injury (PBBI)-induced neuroinflammatory and neuropathological events.^103^

In 2005, Grotta and co-workers^105^ performed an interesting study in which Glypromate and Trofinetide were tested alone or coadministrated with caffeinol (mixture of caffeine and ethanol) in a rat middle cerebral artery (MCA) suture occlusion model.^105^ The combination of Glypromate and caffeinol failed to demonstrate a superior efficacy compared with these compounds alone. Surprisingly, when coadministrated with caffeinol, Trofinetide exhibited strong beneficial effects on cortical and subcortical lesion size in contrast with the administration of Trofinetide alone.^105^ These findings were patented by Grotta and co-workers in the same year.^106^

Svedin and co-workers reported that Trofinetide can moderately attenuate neuronal injury, cause changes in inflammatory markers, and promote astrogliosis in postnatal Wistar rats.^107^ This study indicates that the administration of Trofinetide (1.2 mg kg^–1^ once a day for 7 days, starting 2 h after HI insult) statistically reduces the extension of brain injury in several regions of the immature rat brain (compared with the control group in which a saline injection 2 h after HI insult was given), including CA1/2 (2.76 ± 0.22 vs 3.32 ± 0.11, *p* = 0.0388), CA3 (3.03 ± 0.25 vs 3.64 ± 0.09, *p* = 0.0268), and thalamus (1.60 ± 0.14 vs 2.00 ± 0.07, *p* = 0.0096).^107^ The total neuropathological score was statistically lower for Trofinetide-treated animals than for the control group (2.37 ± 0.25 vs 2.87 ± 0.28, *p* = 0.0240).^107^ The expression of IL-1β and IL-18 was exacerbated in the ipsilateral hemisphere of both Trofinetide-treated and control group animals in comparison with the contralateral hemisphere (IL-1β: 5.3-fold increase in Trofinetide-treated animals and 6.1-fold increase in the control group; IL-18: 1.9 increase in both groups), no significative differences were observed between the Trofinetide-treated animals and the control group in the ipsilateral hemisphere (*p* = 0.9539).^107^ However, for IL-6, a statistical reduction of 32% was observed in Trofinetide-treated animals in the ipsilateral hemisphere (51.0 ± 4.7 pg mg^–1^ compared to 74.8 ± 7.8 pg mg^–1^ in the control group, *p* = 0.0205).^107^ In addition, the treatment with Trofinetide significantly increased the GFAP-positive cell density (22.6 ± 1.8, *p* < 0.001) in the hippocampus compared to the control group.^107^ Although conflicting results were observed for inflammatory response changes and astrogliosis, the authors consider that Trofinetide may find application in the development of effective neuroprotective compounds to treat newborns suffering from birth asphyxia since perinatal HI accounts for acute chronic neurologic morbidity and mortality in infants and children.^107^

In a different study, Lu and co-workers verified that Trofinetide is able to successfully inhibit injury-induced nonconvulsive seizures (64% of the animals at a dose of 100 mg kg^–1^).^108^ The same authors studied the neuroprotective effects of Trofinetide in a rat model of PBBI, demonstrating that this analog exhibits antiapoptotic and antineuroinflammatory activity.^103^

**Figure 11:**
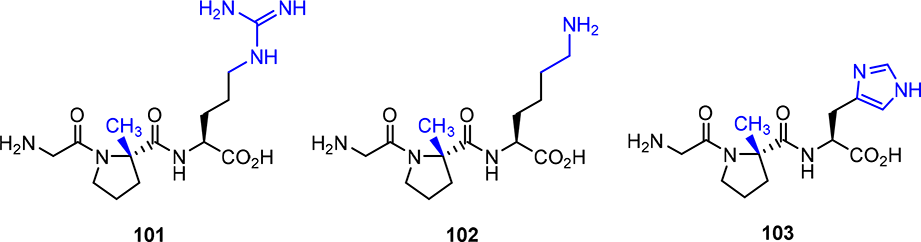
Trofinetide analogs with anti-inflammatory activity.^109^

Despite the great pharmacological potential of Trofinetide, there are only a few reported Trofinetide-based analogs. Considering the great performance of compound **66** (Figure 6), Cacciatore and co-workers synthesized a series of analogs of this peptidomimetic by replacing proline with α-methylproline (compound **101**, Figure 11) and further explored the replacement of arginine by other basic amino acids such as lysine (compound **102**) and histidine (compound **103**), as highlighted in Figure 11.^109^

All of these derivatives enhanced plasma stability (*t*_1/2_ > 51 h) in comparison with **66** (*t*_1/2_ = 1.54 h).^109^ Notably, compound **103** was the most active compound of the series, denoting a superior performance compared with **66**.^109^ Neuroprotection assays were carried out in SH-SY5Y cells using the secretion of human THP-1 cells stimulated with a combination of inflammatory mediators, like IFN-γ and LPS as the toxicity insult or dexamethasone (1 μM), and the cell viability was determined by the LDH assay.^109^ When compounds **101**–**103** (100 μM) were used 1 h before incubation with conditioned medium for 24 h, the cell viability was statistically reduced between 70 and 84% in comparison with the control condition.^109^ However, no cell viability values were provided for each condition. The effect of compounds **101**–**103** on nitric oxide (NO) production was investigated in SH-SY5Y cells. At the highest concentration used (100 μM), all compounds were found to statistically (*p* < 0.05) reduce the NO production with respect to the control.^109^ In this series, compound **103** was the most promising analog, reducing the NO production from 3.75 ± 0.14 to 2.38 ± 0.11 μmol NO L^–1^/10^6^ cells (*p* < 0.05) when compared to **66** (2.64 ± 0.13 μmol NO L^–1^/10^6^, *p* < 0.05).^109^ However, the graphical representation of these data in the original article does not appear to reflect this observation with compound **102** being claimed by the authors as the most promising, which suggests incompatibility between the values provided in the support information and the graphic provided in the paper. Complementary assays demonstrate that when SH-SY5Y cells are incubated with THP-1 conditioned medium, the activity of inducible NO synthase (iNOS) is substantially increased (*p* < 0.01),^109^ while a decrease in neuronal NO synthase (nNOS) activity (*p* < 0.01) is also verified.^109^ Both effects were countered by pretreatment with **101**–**103** for 1 h.^109^ Changes in NOS activity were accompanied by alterations in protein expression (normalized to β-actin levels) as demonstrated by Western blot analyses.^109^ Interestingly, compound **103** was able to statistically return the nNOS protein levels close to the control (*p* < 0.05).^109^ The effect of the NF-κB transcription factor on the cytokine-mediated neuronal activation was then studied via the protein levels of NF-κB inhibitor-α (IkBA) and phosphorylated protein p65 (RelA).^109^ It was shown by Western blot that the conditioned medium induced a significant increase of cytoplasmatic phosphorylated IkBA (*p* < 0.05, normalized to IkBA levels) in SH-SY5Y cells.^109^ This effect was completely reverted by gliotoxin, a selective NF-κB inhibitor, and a similar effect was verified for **103** (100 μM).^109^ As well, it was noted an increase in nuclear phosphorylated protein RelA (*p* < 0.05, normalized to β-tubulin levels), which was significantly reverted by **103** and gliotoxin (*p* < 0.05).^109^ Based on these results, compound **103** was able to suppress the NF-κB-mediated inflammatory response.^109^ The stabilization of IkBA appears to be a plausible explanation for the reduction of NO levels.^109^

## 4 Overview and Conclusions

**Figure 12:**
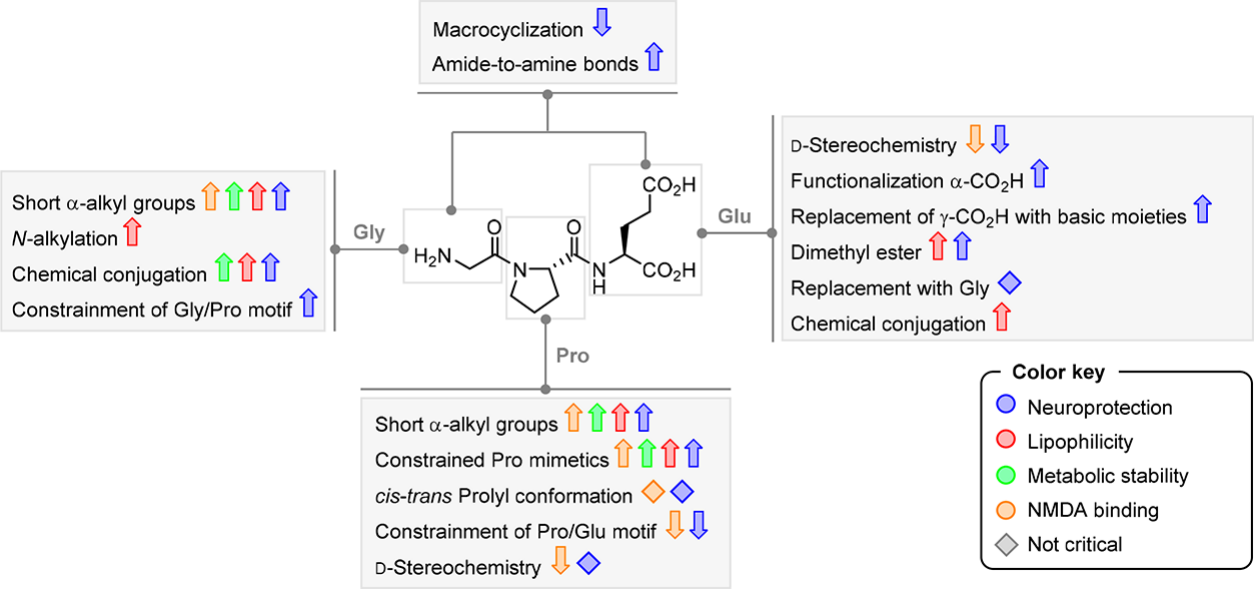
Summary of the main structure–activity relationship results gathered from the data obtained in the literature and reviewed in this work.

Glypromate displays a multitude of biological responses within the CNS exhibiting interesting neuroprotective activity in many *in vitro* and *in vivo* models of neurodegenerative diseases and other neurological conditions. However, the underlying mechanisms remain not fully understood. Despite its potential clinical application in the treatment of many CNS-related disorders, this neuropeptide has major pharmacokinetic drawbacks, such as low stability in plasma and low membrane permeability, hampering its oral administration. Over the past 20 years, many efforts have been made not only to leverage and optimize the neuroprotective profile of Glypromate but also to tower over the unfavorable pharmacokinetics associated with this small peptide. The development of different classes of peptidomimetics has provided interesting structure–activity relationship insights and promising results toward the optimization and implementation of Glypromate-based neuroprotective drugs. The main structure–activity relationship results are summarized in Figure 12.

Concerning glycine mimetics, the introduction of small alkyl groups (e.g., methyl) at either the α-carbon or the amino group of the glycine residue of Glypromate seems to improve neuroprotection and the displacement of [^3^H]-l-glutamate in binding assays,^4,53^**2** and **12** being two paradigmatic examples of well-succeeded Glypromate analogs. The replacement of glycine by alanine, although considered a minor chemical modification, has been found to improve lipophilicity, metabolic stability, and neuroprotection performance when compared with the parent neuropeptide. More importantly, the preference for *S*-stereochemistry of alanine (l-alanine, analog **2**) seems to be a structural requirement since neuroprotection is abolished when the d-alanine counterpart is used instead (**1**). The development of cyclic derivatives at the α-carbon of glycine is also a prolific strategy affording peptidomimetics with enhanced pharmacokinetic profiles (**4** and **5**) when compared to Glypromate. Despite improving the displacement of [^3^H]-l-glutamate, the introduction of acidic (**7**) or basic residues (**8**) as glycine surrogates results in lower neuroprotective activity when compared to the parent neuropeptide. The same trend was observed when aromatic (**6**) and aliphatic residues with longer alkyl chains (**9** and **10**) were used instead. Capping strategies of the N-terminal of Glypromate were found to improve both lipophilicity and neuroprotective activity, with particular emphasis on derivative **12** which displays a *N*,*N*-dimethylglycine residue as a glycine surrogate.

Proline is the most explored residue for the development of Glypromate analogs. The use of constrained proline mimetics generally renders bioactive compounds. Structure–activity relationship studies show that the absolute configuration of proline residue is not deemed as an essential requisite since analog **33** (incorporating a d-proline residue) retains neuroprotection activity. Heterocyclic scaffolds such as thiazolidines **21** and **22** were found to display low affinity toward NMDA receptors but high neuroprotective activity, in particular analog **22**. The introduction of a sulfur atom, i.e., the conversion of pyrrolidine into a thiazolidine in **21** and **22**, is hinted to alter the puckering and lipophilicity of the central residue with loss of binding toward NMDA receptors. While **21** is known to exist mostly in the *trans* conformation, the presence of the dimethyl group at the C-5 position in compound **22** is known to cause a shift in the *cis*–*trans* equilibrium in favor of the *cis* counterpart. Altogether, these results demonstrate that pyrrolidine ring puckering of proline is a key determinant for NMDA binding and neuroprotective effects while *cis*–*trans* prolyl isomerization is not correlated with glutamate receptor binding affinity or neuroprotective activity.

The introduction of polar groups at position 4 of the pyrrolidine moiety (**25** and **26**) seems to increase the binding affinities toward NMDA receptors albeit these results do not translate into improved neuroprotective activity.

Among prolyl-based Glypromate analogs, the results suggest that increased lipophilicity, ring constriction, or the association of both factors (e.g., **15**, **28**, and **41**) results in enhanced neuroprotective activity. Azanorbornane-based Glypromate analogs (**28** and **41**–**44**), which are chimeric constructs between pyrrolidine and piperidine structures, are considered privileged scaffolds since they can leverage the neuroprotective activity in comparison with Glypromate and analog **30**. To date, the most successful analog described in the literature is Trofinetide (compound **15**), corroborating that constriction and lipophilicity at central residue can imprint a great impact on the bioactivity and pharmacokinetics of this neuropeptide. The replacement of the α-proton of proline residue by a methyl group effectively improves oral bioavailability and protection against enzymatic degradation, with improved anti-inflammatory and antiapoptotic properties.^57^

Considering the chemical modifications carried out at the glutamic acid, the conversion of carboxylic acids to *N*,*N*-dimethylamides appears to be site-sensitive. While *N*,*N*-dimethylamide at α-position (**50**) exhibits 20–40% recovery between 1 and 100 mM, the neuroprotection is lost at the side chain (γ-position, **57**). Additionally, the reduction of carboxylic acids at either α- or γ-positions into the corresponding alcohols, **51** and **58**, respectively, is known to abolish (**51**) or induce a significantly lower neuroprotective effect (**58**). Capping strategies such as the conversion of the carboxylic acids into short alkyl esters (e.g., **60**) is an important approach to improve lipophilicity with partial retention of the neuroprotective activity. Contraction (e.g., aspartate-based analogs **62** and **63**) or elongation (e.g., homoglutamic acid-based analogs **64** and **65**) of the side chain abolishes the affinity for glutamate receptors. Analog **66**, which features an arginine residue as a glutamic acid surrogate was the one with the most promising antiapoptotic properties and potential application in AD.^69^ Together with results obtained for compounds **101**–**103** (Trofinetide analogs), which exhibit basic residues as glutamic acid surrogates, the replacement of γ-carboxylic acid with basic scaffolds indicates enhanced neuroprotective effects.

Interestingly, in binding experiments at the NMDA receptor using [^3^H]-l-glutamate, it was shown that the *S*-stereochemistry plays a pivotal role in glutamate residue since the epimer of Glutamate (**53**), which possesses the d-configuration, is not able to displace the binding of [^3^H]-l-glutamate.^61^ Despite being a key determinant for NMDA receptor binding, the glutamate residue is not deemed as essential for neuroprotective activity since its replacement by glycine (compound **59**) is known to retain neuroprotective effects when compared to Glypromate.

Other strategies such as cyclization have shown that Gly-Pro rigidification seems to improve neuroprotective activity at subnanomolar concentration, with **69** and **71** being two successful examples in this regard. Amide-to-amine replacement (**76**–**78**) was demonstrated to improve resistance toward proteolysis without compromising antineuroinflammatory activity.

Recently, the chemical conjugation of Glypromate dimethyl ester (**60**) with lipoic acid and peracetylated L/D configuration-DOPA was shown to be a prolific strategy to explore synergistic effects, affording conjugates **80** and **81**, respectively. While conjugate **80** was found to exhibit neuroprotective activity (*in vitro*) using a cellular model of AD improving the Aβ-induced AChE activity in comparison with Glypromate, conjugate **81** exhibited neuroprotective effects in an animal model of PD. Moreover, Glypromate-based nanomaterials, such as **88**, demonstrated promising neuroprotective effects in animal models of AD.

The chemistry of Glypromate analogs and the biological outcomes of the peptidomimetics described in this review are rich and expected to provide useful structure–activity relationship insights for the rational design of valuable neuroprotective compounds, opening new avenues for the discovery of new neurotherapeutics to manage neurodegenerative conditions of the CNS.

## Abbreviation list

6-OHDA: 6-hydroxydopamine
Aβ: amyloid β
AChE: acetylcholinesterase
AD: Alzheimer disease
Aib: α-aminoisobutyric acid
AMPA: α-amino-3-hydroxy-5-methyl-4-isoxazolepropionic acid
Akt: protein kinase B
API: active pharmaceutical ingredient
APV: d-2-amino-5-phosphonovalerate
BBB: blood–brain barrier
BDNF: brain-derived neurotrophic factor
CGI-I: clinical global impression-improvement
cGP: cyclo-glycyl-l-proline
ChAT: choline acetyltransferase
CNS: central nervous system
CNQX: 6-cyano-7-nitroquinoxaline-2,3-dione
CREB: cAMP response element binding protein
CSF: cerebrospinal fluid
DIC: *N*,*N*-diisopropylcarbodiimide
EC_50_: half-maximal effective concentration
EDC: 1-[3-(dimethylamino)propyl]-3-ethylcarbodiimide
E-factor: environmental factor
ERK: extracellular signal-regulated kinase
FDA: Food and Drug Administration
GABA: γ-aminobutyric acid
GAD: glutamate decarboxylase
GAL: galantamine
GPE: glycyl-l-prolyl-l-glutamic acid
GQD: graphene quantum dots
GSH: striatum glutathione
GSK-3β: glycogen synthase kinase-3β
HI: hypoxic–ischemic
HO-1: heme oxygenase 1
HOBt: 1-hydroxybenzotriazole
IkBA: NF-κB inhibitor-α
ip: intraperitoneal
IGF-1: insulin-like growth factor-1
IL: interleukin
LA: lipoic acid
LDH: lactate dehydrogenase
L/D configuration-DOPA: levodopa
LPPS: liquid-phase peptide synthesis
LPS: lipopolysaccharide
MAPK: mitogen-activated protein kinase
MCA: middle cerebral artery
MePEGMA: poly(ethylene glycol) methyl ether methacrylate
NF-κB: nuclear factor κB
MK-801: dizocilpine
MMP-2: gelatinase A
MMP-9: gelatinase B
MPTP: 1-methyl-4-phenyl-1,2,3,6-tetrahydropyridine
MTT: 3-[4,5-dimethylthiazol-2-yl]-2,5-diphenyltetrazolium bromide
NADPHd: nicotinamide-adenine dinucleotide phosphate diaphorase
NGF: nerve growth factor
NHS: *N*-hydroxysuccinimidyl
NMDA: *N*-methyl-d-aspartate
NPC: neural progenitor cell
ODD: orphan drug designation
PAMPA: parallel artificial membrane permeability assay
PBBI: ballistic type traumatic brain injury
PBS: phosphate buffered saline
PD: Parkinson’s disease
PEG: polyethylene glycol
PI3K: phosphoinositide 3-kinase
PMA: phorbol 12-myristate 13-acetate
PyBOP: [(1*H*-benzotriazol-1-yl)oxy]tri(pyrrolidin-1-yl)phosphonium hexafluorophosphate
RelA: protein p65
RSBQ: Rett syndrome behavior questionnaire
SPPS: solid-phase peptide synthesis
SRIF: somatostatin
TAC: total antioxidant capacity
TFA: trifluoroacetic acid
TFE: 2,2,2-trifluoroethanol
TH: tyrosine hydroxylase
ThT: thioflavin-T
TNF-α: tumor necrosis factor-α
TOS: total oxidative status
